# Transcriptomic disruption and hypoactivity in DYT-SGCE medial ganglionic eminence-patterned inhibitory neurons

**DOI:** 10.1093/brain/awaf272

**Published:** 2025-07-25

**Authors:** Zongze Li, Laura Abram, Maria Cruz-Santos, Olena Petter, Kathryn J Peall

**Affiliations:** Division of Psychological Medicine and Clinical Neurosciences, Cardiff University, Cardiff CF24 4HQ, UK; Neuroscience and Mental Health Innovation Institute, Cardiff University, Cardiff CF24 4HQ, UK; Division of Psychological Medicine and Clinical Neurosciences, Cardiff University, Cardiff CF24 4HQ, UK; Neuroscience and Mental Health Innovation Institute, Cardiff University, Cardiff CF24 4HQ, UK; Division of Psychological Medicine and Clinical Neurosciences, Cardiff University, Cardiff CF24 4HQ, UK; Neuroscience and Mental Health Innovation Institute, Cardiff University, Cardiff CF24 4HQ, UK; Division of Psychological Medicine and Clinical Neurosciences, Cardiff University, Cardiff CF24 4HQ, UK; Neuroscience and Mental Health Innovation Institute, Cardiff University, Cardiff CF24 4HQ, UK; Division of Psychological Medicine and Clinical Neurosciences, Cardiff University, Cardiff CF24 4HQ, UK; Neuroscience and Mental Health Innovation Institute, Cardiff University, Cardiff CF24 4HQ, UK

**Keywords:** dystonia, neurons, stem cells, disease modelling

## Abstract

Myoclonus dystonia is a Mendelian inherited, childhood-onset dystonic disorder, caused by mutations in the autosomal dominantly inherited gene *SGCE*, and in which both motor and psychiatric phenotypes are observed. Results from murine and *in vivo* human studies suggest that dystonia is caused by disruption to neuronal networks, in particular the basal ganglia–cerebello-thalamo-cortical circuit. Work focused on the cortical component implicates disruption to neuronal excitatory–inhibitory balance as being a key contributor to the observed phenotypes. Our previous work, focused on cortical excitatory glutamatergic neurons, demonstrated a hyperexcitable phenotype and more complex dendritic arborization in an *in vitro* model of myoclonus dystonia. In contrast, human electrophysiological studies have suggested that it is the loss of inhibitory tone in this region that contributes to the overall hyperkinesis. To explore this further, we have evaluated the impact of *SGCE* mutations on medial ganglionic eminence-derived inhibitory GABAergic neurons using the same patient-derived induced pluripotent and gene-edited embryonic stem cell lines, comparing each with their isogenic wild-type control.

Differentiation towards inhibitory interneurons demonstrated no significant differences in either early stage (NKX2.1 and FOXG1) or late stage (GAD67 and GABA) developmental markers. Single-cell RNA sequencing also confirmed evidence of markers consistent with medial ganglionic eminence-derived GABAergic neurons and, when compared with two publicly available human fetal ganglionic eminence transcriptomic datasets, confirmed that the cells generated resembled those found *in vivo*. Further analysis of these data demonstrated transcriptomic dysregulation in genes related to axonal organization, synaptic signalling and action potential generation in the *SGCE*-mutation-positive neurons. Subsequent characterization of dendritic morphology found *SGCE*-mutation-positive neurons to have shorter branches, fewer higher-order branches and reduced branching complexity, in comparison to their wild-type controls. Functional analyses using Ca^2+^ imaging and multi-electrode array approaches to examine network activity identified significantly lower calcium responses to GABA and reduced spike and burst frequencies in the *SGCE*-mutation-carrying lines, in comparison to their isogenic controls. Reduced activity was also observed in single-cell patch-clamp studies, with fewer neurons firing action potential trains, coupled with fewer spontaneous postsynaptic currents, in comparison to controls.

Collectively, this work indicates lower neuronal inhibitory activity and complexity of the dendritic arbor in the context of *SGCE* mutations, further contributing to the disruption of neuronal excitatory–inhibitory balance in motor circuits and potentially underlying the observed clinical hyperkinetic phenotype. These changes might also represent common characteristics across the wider dystonia spectrum, with potential for future target identification with amenability to therapeutic intervention.

## Teaser

Myoclonus dystonia is a movement disorder caused by mutations in the *SGCE* gene. Li *et al.* used patient-derived stem cells to generate GABAergic inhibitory neurons with an *SGCE* mutation. These neurons were less active and had simpler branching patterns than control neurons, shedding light on the cellular consequences of the mutation.

## Introduction

Dystonia is one of the most common forms of movement disorder, with an estimated population prevalence of 1.2%.^1,2^ It involves loss of coordinated contraction of antagonistic muscle groups, leading to abnormal postures and pain, with subsequent impact on quality of life.^3^ The clinical presentation of dystonia is heterogeneous, involving single or multiple muscle groups (focal, segmental or generalized), genetic or idiopathic in aetiology, and of childhood or adult onset.^4,5^ Co-morbid psychiatric symptoms are also observed, frequently pre-dating onset of the motor symptoms and spanning a spectrum of anxiety, obsessive–compulsive disorder, depression and social phobia.^6^

In excess of 50 Mendelian-inherited dystonia-causing genes have now been identified, with these predominantly resulting in onset of motor symptoms in early life stages.^7^ One such disorder is myoclonus dystonia, caused by mutations in the autosomal dominantly inherited gene *SGCE*, encoding the protein ε-sarcoglycan.^8,9^ The associated clinical phenotype typically involves upper-body-predominant myoclonus, focal or segmental dystonia involving the cervical and/or upper limb regions, and psychiatric symptoms including generalized anxiety disorder and obsessive–compulsive disorder.^10,11^ The ε-sarcoglycan protein is a single-pass transmembrane glycoprotein,^12^ which is expressed embryonically and postnatally, suggesting its importance in development.^13^ Examination of the brain-specific form of ε-sarcoglycan suggests that it forms part of a brain-specific dystrophin-associated protein complex,^14^ with ultra-deep sequencing of post-mortem brain tissue demonstrating high levels of expression in the primary somatosensory cortex.^15^

Understanding of the pathophysiological mechanisms underpinning dystonia remains limited; however, evidence from human imaging, murine and post-mortem studies indicates a disruption to neuronal networks, principally involving the basal ganglia–cerebello-thalamo-cortical circuits.^16,17^ Human imaging studies have identified white and grey matter morphometric and white matter microstructural changes in regions linked with the cerebral cortex,^18-21^ and electrophysiological studies, in humans and mice, have demonstrated increased cortico-striatal long-term potentiation and reduced long-term depression in the motor cortex.^22-24^ Several studies have suggested that impaired cortical surround inhibition is responsible for these changes; however, recent work has suggested a more complex picture, with additional factors contributing to the hyperexcitable phenotype.^24,25^ Our recent work involving Clustered Regularly Interspaced Short Palindromic Repeat (CRISPR)-edited embryonic stem cell and patient-derived induced pluripotent stem cell (iPSC) models of *SGCE*-mutation-positive myoclonus dystonia differentiated towards an excitatory glutamatergic cortical neuronal lineage, demonstrated a hyperexcitable phenotype, more complex dendritic branching morphology and disruption to the synaptic adhesion molecules neurexin-1 and neuroligin-4.^2^

In this study, we seek to build on this work through differentiation of the same stem cell models towards a medial ganglionic eminence (MGE)-like-derived inhibitory neuronal lineage, the most common subtype of inhibitory neurons in the cerebral cortex, determining the impact of the loss of ε-sarcoglycan expression on their development, structure and function. We aim to determine whether the excess motor activity observed in dystonia is driven purely by the hyperexcitability observed in excitatory glutamatergic cortical neurons or whether, in line with *in vivo* electrophysiological evidence, there is an additional loss of inhibitory activity in MGE-derived GABAergic neurons. Understanding these changes is crucial to identification of new therapeutic targets and future therapeutic development.

## Materials and methods

### Experimental model

Development of both the patient-derived iPSC and CRISPR/Cas9 embryonic stem cell lines has been reported elsewhere.^2^ All participants were examined and confirmed to have a clinical phenotype consistent with myoclonus dystonia. They were recruited to the Welsh Movement Disorders Research Network, giving signed informed consent for derivation of iPSC lines in line with the Declaration of Helsinki (REC for Wales, IRAS ID: 146495, REC ref.: 14/WA/0017). Confirmation of pathogenic *SGCE* variants was provided by National Health Service diagnostic laboratory genetic testing.

### Stem cell culture

The iPSCs and human embryonic stem cells (hESCs) were cultured on Cultrex Stem Cell Qualified Reduced Growth Factor Basement Membrane Extract (R&D Systems) and maintained in Essential 8 flex medium in standard culture conditions (37°C, 5% CO_2_). The stem cell medium was changed on alternate days, and cells were passaged every 3–4 days when 70%–80% confluency was reached. Primary stem cells (PSCs) were mechanically dissociated and re-plated with Gentle Cell Dissociation Reagent (STEMCELL Technologies) for maintenance and cryopreservation or with ReLeSR (STEMCELL Technologies) for neuronal differentiation upon reaching ∼70%–80% confluence.

### MGE-derived GABAergic neuron differentiation

MGE-derived GABAergic neurons were differentiated from PSCs as previously reported.^25^ In brief, differentiation was initiated when the culture reached ∼80% confluence within 2 days of replating. On Day 7 of ESC differentiation or Day 9 of iPSC differentiation, cells were mechanically dissociated using 0.5 mM EDTA and replated at a ratio of 1:1–1:1.5 by area. On Days 21 and 30, cells were dissociated to single cells and plated at a density of 5 × 10^5^ and 1.25 × 10^5^ cells/cm^2^, respectively. Y27632 (Stratech Scientific; 10 µM) was added to the culture media for the first day after each passage. Neuronal differentiation was promoted by N2B27-RA basal medium supplemented with 100 nM LDN193189 (Sigma-Aldrich), 10 µM SB431542 (Tocris) and 2 µM XAV939 (Stratech Scientific) from Day 0 to 9, 200 ng/ml recombinant SHH (Peprotech), with 1 µM Purmorphamine (Sigma-Aldrich) from Day 10 to 20, and 10 ng/ml brain-derived neurotrophic factor (BDNF; Peprotech) after Day 25. On Day 25, B27 supplement without retinoic acid was replaced by B27 supplement with retinoic acid.

### Immunocytochemistry

Cultured cells were washed with PBS, fixed in cold (4°C) 3.7% paraformaldehyde for 20 min, re-washed three times with PBS and stored at 4°C until required. To allow for immunocytochemistry of intracellular markers, cells were permeabilized by sequential washes with 33% and 66% methanol at room temperature, 100% methanol at −20°C, returned to PBS via an inverse gradient, then blocked in PBS-T (0.3% Triton X-100 in PBS) containing 1% bovine serum albumin and 3% donkey serum at room temperature for 1–3 h. After blocking, cells were incubated with primary antibodies (Supplementary Table 1) in PBS-T, containing 1% bovine serum albumin and 1% donkey serum, at 4°C overnight. The next day, cells were washed with PBS-T as described above and incubated with secondary antibodies (Supplementary Table 1), in darkness, at room temperature for 1 h. The cells were then washed with PBS-T and counterstained with 4′,6-diamidino-2-phenylindole (Molecular Probes) diluted 1:3000 in PBS-T in darkness. Finally, the cells were washed three times with PBS-T and PBS and mounted with a fluorescence mounting medium. Stained cells were imaged using a Leica DMI6000B inverted microscope, with an average of 10 random fields of view for each staining combination at ×20 magnification and images processed using LAS X software (Leica). Automated cell quantification was carried out using Cell Profiler v.4.2.7.^26^ Data for immunocytochemical quantification were collected from at least three biological replicates, from at least three independent experiments, for each marker.

### Quantitative real-time PCR

Total RNA was extracted using TRI reagent, treated with DNase I and cDNA generated using EvoScript Universal cDNA Master Version 04 (Roche). Quantitative real-time PCR (qPCR) was performed using Takyon™ Low ROX Probe 2× MasterMix dTTP blue to quantify genes of interest. The Applied Biosystems QuantStudio™ 7 Flex Real-Time PCR System was used for the standardized qPCR programme. The PCR run involved: increase to 50°C (1.6°C/s), 50°C (2 min), increase to 95°C (1.6°C/s), 95°C (10 min). The PCR stage involved a total of 45 cycles, each 95°C (15 s), decreasing to 60°C (1.6°C/s), and held at 60°C (1 min) for fluorescence data collection. Dissociation curves were recorded to check for amplification specificity. Relative quantification was determined using the ΔΔ−*C*_T_ method with QuantStudio Real-Time PCR software v.1.3. All data were normalized to the geometric means of *C*_T_ values of two endogenous control genes (*GAPDH* and *ACTB*). Reactions were performed in triplicate for each cDNA sample, for each of the three differentiations. Primers are listed in Supplementary Table 2.

### Single-cell RNA sequencing

Day 80 neuronal cultures were dissociated with Accutase (8 min at 37°C), resuspended in Dulbecco's phosphate buffered saline (DPBS), mixed with 0.5% bovine serum albumin and 50 units/ml of DNase I (Sigma Aldrich), and passed through a 35 µm mesh cell strainer. Cells were stained with the ReadyProbes™ Cell Viability Imaging Kit, Blue/Red (Invitrogen) following the manufacturer's guidance, and dispensed into the nano-well plates of the ICELL8^®^ cx Single-Cell System (Takara). Wells containing a single nucleus were automatically and manually selected using the ICELL8 cx CellSelect v.2.5 Software. The sequencing library was prepared using the SMART-Seq ICELL8 application kit (Takara) following the manufacturer's instructions. Next-generation sequencing was performed using the NovaSeq 6000 SP Reagent Kit v.1.5 (200 cycles) on an SP flow cell on NovaSeq 6000 (Illumina).

### Single-cell RNA sequencing analysis

Raw sequencing data were converted into FASTQ files containing all indices for each chip, demultiplexed using the *cogent demux* command and analysed using the *cogent analyze* command, as part of the Cogent NGS Analysis Pipeline software v.2.0.0 (Takara). Sequences were aligned to the *Homo sapiens* GRCh38.94 primary assembly. The count matrix of each sample was combined in R v.4.4.0.^27^ Downstream analysis was performed in R v.4.4.0 using Seurat v.5.1.0^28^ unless otherwise stated. Only protein-coding genes with at least five total counts and expressed in at least eight cells (1%) were included. After gene-level filtering, only cells with >2 × 10^4^ total gene counts, >5000 genes detected and <10% mitochondrial gene count were included for downstream analysis.

Raw counts were normalized using the *NormalizeData* function with a scale factor of 1 × 10^4^ and scaled with the *ScaleData* function, regressing out the percentage of mitochondrial gene count. The top 2000 most highly variable genes, identified using the *FindVariableFeatures* function, were used for principal component analysis (*RunPCA* function). The first 20 principal components were used for downstream integration and dimensional reduction, using the Jackstraw method^29^ and visual inspection of the elbow plot. Data from distinct cell lines were integrated using the *IntegrateLayers* function involving four methods (canonical correlation analysis, Harmony, reciprocal principal component analysis and fast mutual nearest neighbours correction) and yielded similar results. The Harmony method was selected to facilitate standardized analysis with other publicly available datasets.^30^ Uniform manifold approximation and projection (UMAP) and unbiased Louvain clustering was performed using *RunUMAP*, *FindNeighbors* and *FindClusters* functions, using the first 20 components of the Harmony dimensional reduction matrix. Differential gene expression analysis was performed using the *FindMarkers* function with the Wilcoxon rank sum test and Bonferroni correction.

Gene Ontology (GO) enrichment was performed using *enrichGO* function in the clusterProfiler v.4.12.0 package. The *Homo sapiens* GO term database was downloaded using the org.Hs.eg.db v.3.19.1 package, and rrvgo v.1.16.0 was used to group the representative GO terms using the *calculateSimMatrix* function. Overlaps between gene sets were analysed using the *enricher* function in the clusterProfiler v.4.12.0 package. Reference mapping was performed using the *FindTransferAnchors* and *TransferData* functions in Seurat based on the first 30 dimensions of the Harmony loadings from the reference dataset. Reference datasets were downloaded from NCBI's Gene Expression Omnibus and processed in R v.4.4.0 using the same filtering threshold and pipeline as described above. Clusters were re-annotated based on the expression of canonical markers and compared with published annotation where possible.

Gene regulatory networks were inferred based on the filtered non-zero raw count using SCENIC v.1.3.1 in R v.4.3.0. Transcription factor binding motifs were searched within 500–5000 bp up- and downsstream of the transcription start site in the *Homo sapiens*—hg38—refseq_r80—v9 databases downloaded from https://resources.aertslab.org/cistarget/databases/. The regulon enrichment score (area under the curve) calculated by SCENIC was used for differential enrichment of regulons and analysed using the Wilcoxon signed-rank test with Bonferroni correction.

Weighted correlation network analysis was performed using the hdWGCNA v.0.3.01 package.^31-33^ Metacells were created based on seurat_clusters, genotype and sample variables. Neuron1 population was used as the group of interest, and different genotypes were harmonized during the module eigengene calculation. Differential module eigengene analysis was performed using the Wilcoxon rank sum test with Bonferroni correction. Gene networks were visualized and analysed in Cytoscape v.3.10.2 with the igraph v.2.0.3 package.^34,35^ Protein–protein interaction network data were downloaded from BioGRID v.4.4.236, IntAct v.247, String v.12.0, Reactome release 89 and BioPlex v.3, (all accessed on 18 August 2024) and integrated by keeping only unique interaction pairs.^36-41^

### FLIPR calcium assay

On the day of recording, an equal volume of FLIPR™ Calcium 6 Assay buffer was added to cells and incubated for 2 h at 37°C. Drug assay buffers were prepared in Hank's balanced salt solution (HBSS) with Ca^2+^ and Mg^2+^ with 20 mM HEPES buffer at 5× concentration. After incubation, cells were imaged on a FLIPR Penta system (Molecular Devices) at a frequency of 2 Hz for 1 min prior to injection. The drug assay buffers (12.5 µl) were dispensed at 12.5 µl/s and images captured at 2 Hz for a further 5 min. Raw fluorescence intensity data were exported and analysed in R v.4.4.0. The average baseline fluorescence intensity for each well was calculated by averaging the raw fluorescence intensity measured prior to drug application. The normalized change in fluorescence intensity above the baseline (Δ*F*/*F*_0_) for each well was calculated using the following formula:


$$\Delta F/F_{0}=\frac{F_{t}-F_{0}}{F_{0}}$$

where *F_t_* is the fluorescence intensity at time point *t*, and *F*_0_ is the average fluorescence intensity of the baseline period (30 s prior to drug application).

### Neurite tracing assay

Day 80 neuronal cultures were sparsely transfected with 80 ng/cm^2^ pmaxGFP™ Vector (Lonza) using Lipofectamine™ 3000 Transfection Reagent (Invitrogen). Lipofectamine (0.325 µl per well) was used in a final volume of 25 µl per well for 48-well plates. Forty-eight hours after transfection, cells were incubated with NucBlue™ Live ReadyProbes™ Reagent (Hoechst; Invitrogen) in the dark and washed three times with DPBS. Fresh N2B27 medium, without Phenol Red, was added for live-cell fluorescence microscopy using the Leica DMI6000B inverted microscope. After this, cells were fixed and immunolabelled (GABA and 4′,6-diamidino-2-phenylindole), as outlined above, to ensure tracing of relevant cell lines. Neurites were semi-automatically traced using the SNT toolbox (Fiji ImageJ).^42,43^ Strahler and Sholl analyses were performed using the SNT toolbox.

### Multi-electrode array

Day 30 neurons were plated at a density of 1.25 × 10^5^ cells/cm^2^ onto CytoView multi-electrode array (MEA) 24 plates (Axion Biosystems) coated with Cultrex, in N2B27 basal medium containing 10 ng/mL BDNF and 10 µM Y27632. The next day, 1 ml per well of BrainPhys (STEMCELL Technologies) with 10 ng/ml BDNF was added and continued throughout the recording period. Spontaneous extracellular recordings were obtained using the Axion Maestro Pro system at a sampling rate of 12.5 kHz, 3 kHz Kaiser window, 200 Hz infinite impulse response (IIR), and spike detectors. Cultures were maintained in a 37°C and 5% CO_2_ environment during the recording. Recordings were made three times a week on alternate days from Day 36 to 79 of differentiation. Activity metrics were calculated based on the list of spikes, bursts and network bursts, and processed in R v.4.4.0. Electrodes with <1 spike/min were omitted.

### Whole-cell patch clamp

Day 30 neurons were plated and cultured on Cultrex-coated glass coverslips, with neurons assayed between days 78 and 82 of differentiation. Immediately before recording, coverslips were transferred to a recording chamber on an Olympus BX61W differential interference contrast microscope and continuously perfused at 1.5∼2.5 ml/min with extracellular solution (145 mM NaCl, 10 mM D-glucose, 10 mM HEPES, 3 mM KCl, 2 mM MgCl_2_ and 1.25 mM CaCl_2_, adjusted to pH = 7.37–7.40 with NaOH, 305–310 mOsm/L), heated to 35°C. Recordings were performed using MultiClamp 700B amplifier, 5–7 MΩ resistance pipettes, filled with intracellular solution (120 mM potassium gluconate, 10 mM phosphocreatine disodium, 10 mM HEPES, 4 mM adenosine 5′-triphosphate disodium, 2 mM MgCl_2_, 2 mM EGTA dipotassium and 0.5 mM CaCl_2_, adjusted to pH = 7.30 with KOH, and 289–293 mOsm/L). Electrophysiological data were sampled at 20 kHz and filtered at 3 kHz using a Digidata 1550 analog-to-digital converter and pClamp v.10 software. The resting membrane potential was measured in current-clamp mode at 0 pA, with recordings captured immediately after breaking into the cell (within 20 s) to avoid an influence of the internal pipette solution on intracellular ion concentrations. Leak current was subsequently assessed by holding the cell at −60 mV in voltage clamp, with measurement of the residual current once stabilized. Those cells with a leak current >|100 pA| were excluded from onward analysis, as were those with a series resistance >30 MΩ. In current-clamp mode, membrane potential was maintained at −60 mV. Single action potentials were evoked by injecting a 200 pA current pulse for 5 ms, and repetitive action potential firing was evoked by injecting −30 to +70 pA current in 10 pA steps for 1 s. Spontaneous postsynaptic currents were recorded in voltage-clamp mode at −60 mV for an excitatory current or 0 mV for an inhibitory current. Sodium and potassium currents were evoked (−90 to +40 mV in 10 mV steps for 500 ms to determine activation) and a 500 ms 40 mV step to assess inactivation, with continuous perfusion of extracellular solution containing 100 nM tetrodotoxin or 20 mM tetraethylammonium (TEA), respectively. All recording data were analysed in Clampfit v.11.2 and resting membrane potential in R v.4.4.0.

### Statistical analysis

All data were collected from three independent differentiations and presented as the mean ± standard error of mean (SEM) unless otherwise specified. All statistical analyses were performed in R v.4.4.0. Analyses included one-way ANOVA, Wilcoxon signed-rank test, Kruskal–Wallis test and two-sample Kolmogorov–Smirnov test where appropriate. False discovery rate correction was performed for multiple testing. Sholl analysis data, action potential spike response induced by current squares, MEA spike and burst rate were analysed with Poisson regression, zero-inflated Poisson regression and gamma regression models, respectively.

## Results

### Generation of MGE-derived GABAergic neurons

**Figure 1:**
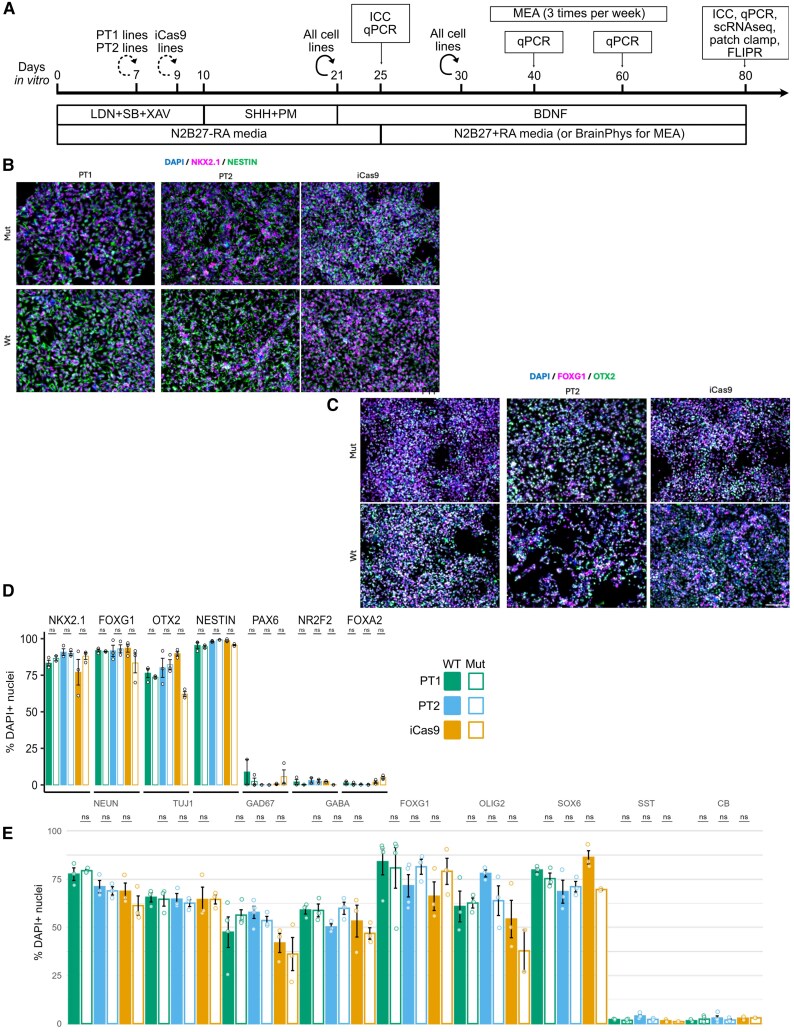
**Absence of impact of *SGCE* mutations on differentiation and developmental markers of medial ganglionic eminence-derived GABAergic neurons.** (**A**) Schematic overview of the differentiation protocol used to derive MGE GABAergic neurons and study design. (**B**) Day 25 differentiation: representative immunofluorescence images for MGE progenitor markers (NKX2.1 and FOXG1), an anterior forebrain marker (OTX2) and a neural stem cell marker (NESTIN). (**C**) Day 25 differentiation quantification of immunofluorescent markers NKX2.1, FOXG1, OTX2, NESTIN and non-MGE lineage markers: PAX6, NR2F2 and FOXA2. Scale bar: 100 μm (**D** and **E**) Day 80 differentiation quantification of immunofluorescent markers of neuronal markers (NEUN and TUJI), GABAergic neuronal markers (GAD67 and GABA), markers of the MGE lineage (FOXG1, OLIG2 and SOX6) and GABAergic neuronal subtypes (SST and CB). Data are presented as the mean ± standard error of the mean from a minimum of three technical replicates from three independent experiments. Lines are compared using Wilcoxon signed-rank tests with false discovery rate correction. ns = not statistically significant (*P* > 0.05); *P < 0.05, **P < 0.01, ***P < 0.001. BDNF = brain-derived neurotrophic factor; ESC = embryonic stem cell; ICC = immunocytochemistry; iPSC = induced pluripotent stem cell; LDN = LDN193189; MEA = multi-electrode array; MGE = medial ganglionic eminence; NPC = neural progenitor; PM = purmorphamine; PT1 = Patient 1; PT2 = Patient 2; RGL = radial glia; SB = SB431542; scRNAseq = single-cell RNA sequencing; SHH = Sonic Hedgehog; XAV = XAV939.

**Figure 2:**
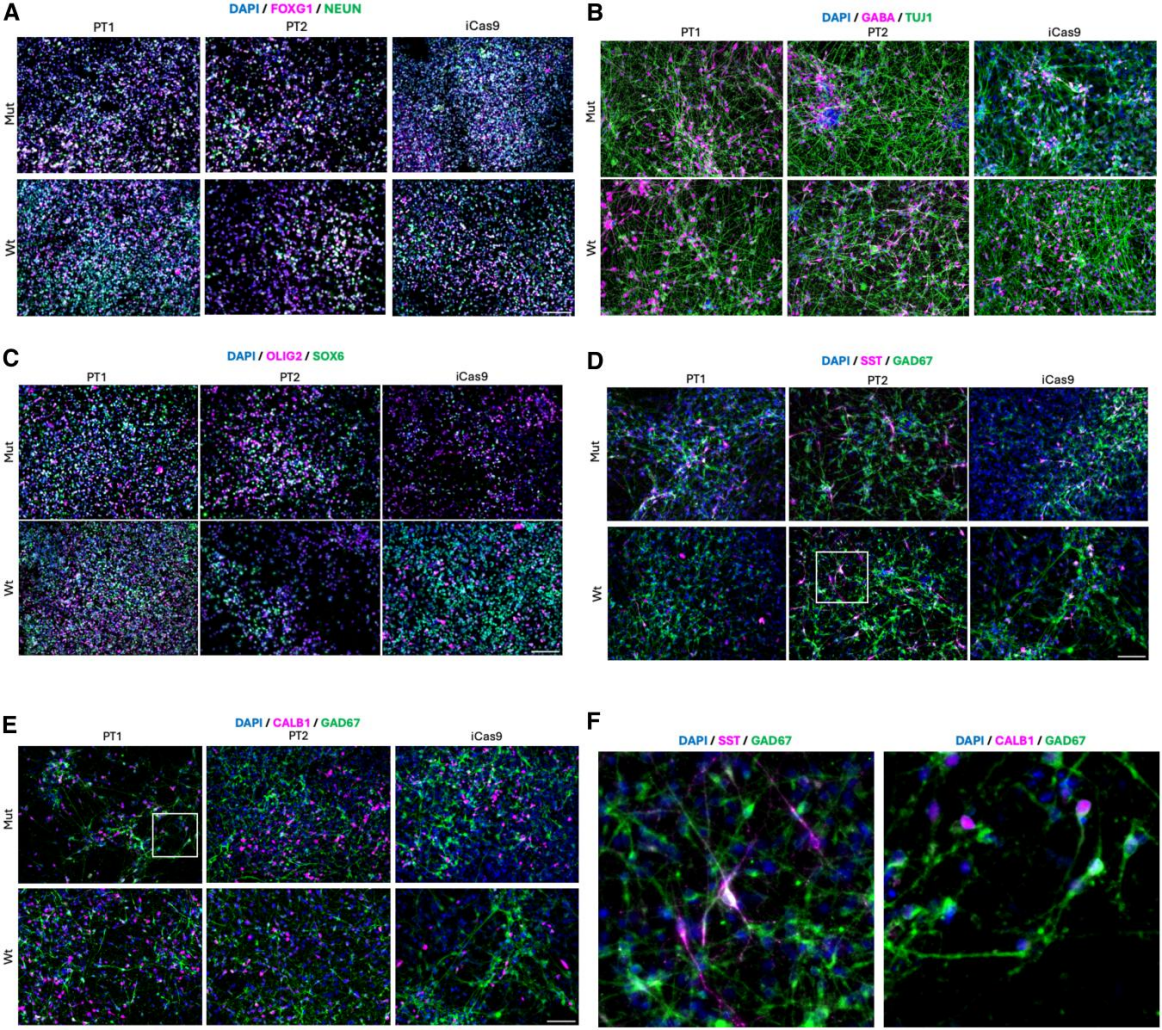
**Medial ganglionic eminence-derived GABAergic neurons later stage developmental markers.** Day 80 differentiation representative immunofluorescence images for: (**A**) FOXG1 and NEUN; (**B**) GABA and TUJ1; (**C**) OLIG2 and SOX6; (**D**) SST and GAD67; and (**E**) CALB1 and GAD67. Scale bars: 100 µm. (**F**) Zoomed views of SST and CALB1 immunofluorescence markers for the two regions highlighted by the white boxes.

To investigate the role of MGE-derived GABAergic neurons in myoclonus dystonia, neurons were differentiated from two patient-derived *SGCE* mutation-positive hiPSC lines (Patients 1 and 2) and a single CRISPR/Cas9-edited *SGCE* knockout iCas9 hESC and their matched isogenic wild-type control lines (Fig. 1A). Immunocytochemical staining at Day 25 of differentiation demonstrated no significant differences in the proportion of cells expressing key MGE progenitor markers, NKX2.1 and FOXG1, anterior forebrain marker, OTX2, and neural stem cell marker, NESTIN, between each cell line pair (Fig. 1B and C). At the same time point, markers of the ventral midbrain (FOXA2), caudal ganglionic eminence (NR2F2), dorsal forebrain and lateral ganglionic eminence (PAX6) were detected in a minimal proportion of cells, with no significant differences between lines (Fig. 1C and Supplementary Fig. 1A). At Day 80 of differentiation, comparable levels of neuronal (NEUN and TUJ1), GABAergic (GAD67 and GABA) and MGE lineage (FOXG1, OLIG2 and SOX6) markers were observed across all cell lines, again with no significant difference between mutant and wild-type lines for each cell line pair (Figs 1D and 2A–E). Some neurons co-expressed SST and CB, suggesting the presence of GABAergic neuron subtypes (Fig. 2F). Further examination of gene expression using qPCR likewise revealed an upregulation of MGE-derived GABAergic neuronal markers (*LHX6*, *GAD67*, *NKX2.1* and *SST*) at Day 80 of the differentiation protocol (Supplementary Fig. 1B).

**Figure 3:**
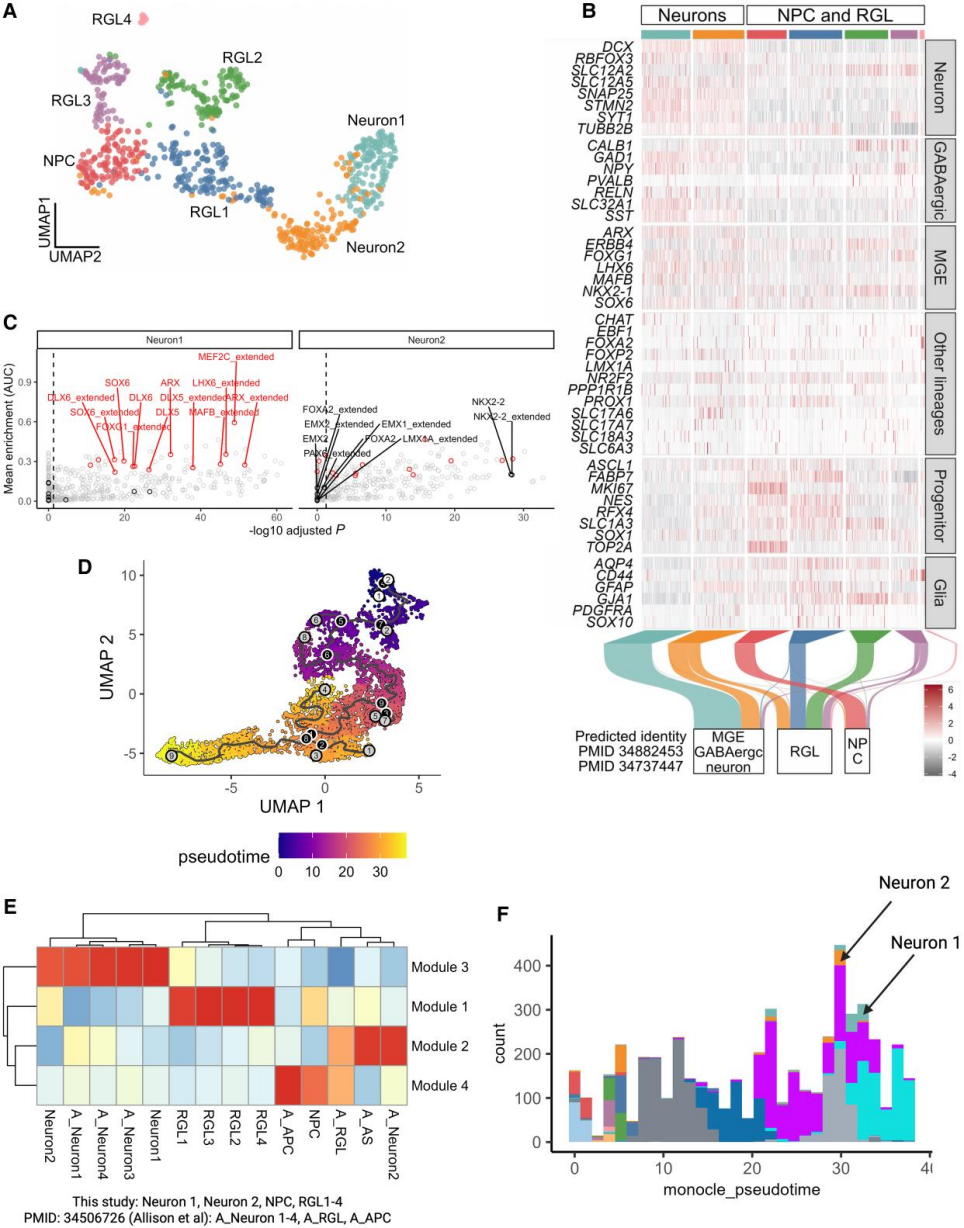
**Transcriptomic support for the derivation of medial ganglionic eminence-derived GABAergic neurons with maturation patterns consistent with *in vivo* derived data.** (**A**) D80 uniform manifold approximation and projection (UMAP) plot of single-cell RNA sequencing (scRNA-seq) of Day 80 wild-type and *SGCE*-mutation-positive MGE-derived GABAergic neurons coloured by cluster annotations. (**B**) Heat map of the expression of canonical marker genes across distinct lineages and cell types, present by cluster. Sankey plot indicating the predicted identity of each cell based on two previously published human fetal MGE scRNA-seq datasets. (**C**) Differentially enriched regulons in neuronal clusters highlighting regulons related to MGE development (red) and non-MGE (black). (**D**) UMAP plot delineating the pseudo-temporal trajectory. Colour gradient references indicate the pseudotime score. (**E**) Averaged expression of gene modules dynamically regulated along the pseudo-temporal trajectory grouped by cell-type clusters identified in this study and previously reported data. (**F**) Histogram representing the pseudotime scores of cells across the integrated pluripotent stem cell-derived dataset, with Neuron 1 and Neuron 2 clusters identified in this study labelled. APC = astrocyte progenitor cell; MGE = medial ganglionic eminence; NPC = neural progenitor; RGL = radial glia; UMAP = uniform manifold approximation and projection.

Data captured from the single-cell RNA sequencing (scRNAseq) analysis were used for further confirmation of cell line genotype and demonstration of markers consistent with MGE-derived GABAergic neurons. Here, *SGCE* mutation-carrying lines demonstrated significantly lower *SGCE* expression, whereas no significant difference was observed in the expression levels of the other members of the sarcoglycan family (Supplementary Fig. 1C). After data integration (Supplementary Fig. 2A), seven clusters of conserved cell types were identified across the two genotypic groups (Fig. 3A and Supplementary Fig. 2B), with the proportions of profiled cells per cell type and genotype detailed in Supplementary Fig. 2C and D. Two clusters (Neuron1 and Neuron2) expressed high levels of pan-neuronal markers (*SYT1*, *STMN2*, *SNAP25*, *RBFOX3* and *DCX*) but low levels of progenitor (*MKI67*, *TOP2A*, *NES* and *SOX1*), radial glia (*SLC1A3*, *RFX4* and *FABP7*) and glial (*SOX10*, *PDGRFA*, *GJA1*, *GFAP*, *CD44* and *AQP4*) markers. In addition, Neuron1 and Neuron2 clusters expressed higher levels of markers for cells of an MGE-derived lineage (*NKX2-1*, *LHX6*, *FOXG1*, *MAFB*, *ERBB4*, *ARX* and *SOX6*) and GABAergic neurons (*GAD1*, *SLC32A1*, *CALB1*, *RELN*, *SST* and *NPY*). There was a near absence of markers for cells of lateral and caudal ganglionic eminence lineage, in addition to glutamatergic, dopaminergic and cholinergic neurons (Fig. 3B). In addition, SCENIC regulon enrichment found Neuron1 and Neuron2 clusters to be differentially enriched for regulons of key transcription factors involved in the specification of MGE-derived GABAergic neurons (including LHX6, SOX6, NKX2.1, FOXG1, ARX, MAFB, MEF2C and DLX5/6) but not transcription factors involved in the development of other neuronal lineages (Fig. 3C).

Two publicly available scRNAseq datasets of human fetal ganglionic eminence tissue were used as reference to ensure that the GABAergic neurons generated resembled those found *in vivo*. Here, cells in clusters Neuron1 and Neuron2 were predicted to resemble MGE-derived GABAergic neurons in both datasets, while neural progenitor (NPC) clusters and the four radial glial clusters mapped to neural progenitors, radial glial and glial populations, respectively (Fig. 3B and Supplementary Fig. 2E). Moreover, integrative analysis revealed that the neurons generated exhibited similar human fetal MGE-derived GABAergic neuron-like transcriptomic identity to PSC-derived MGE-lineage GABAergic neurons observed in previously reported data (Supplementary Fig. 3A–C).^44^ Furthermore, to compare the transcriptomic maturity between PSC-derived MGE-lineage GABAergic neurons generated and those reported by Allison *et al*.,^44^ a pseudotemporal trajectory was constructed (Fig. 3D). Genes upregulated along the trajectory (module 3) included genes related to aspects of neuronal development and maturation (Fig. 3E, Supplementary Fig. 3D and Supplementary material, ‘Data 1'), suggesting that the trajectory represents a neurodevelopmental and maturation axis. Along this trajectory, cells in both neuronal clusters (Neuron1 and Neuron2) demonstrated similar pseudotime maturity scores to previously reported neuronal cultures (Fig. 3F).^44^ Collectively, these data indicate that the PSC-derived neurons in this study demonstrated not only a transcriptomic identity consistent with human fetal and PSC-derived MGE-patterned GABAergic neurons, but also a maturity consistent in keeping with previously reported studies.

### Dysregulated transcriptomic landscape in *SGCE*-mutant GABAergic neurons

**Figure 4:**
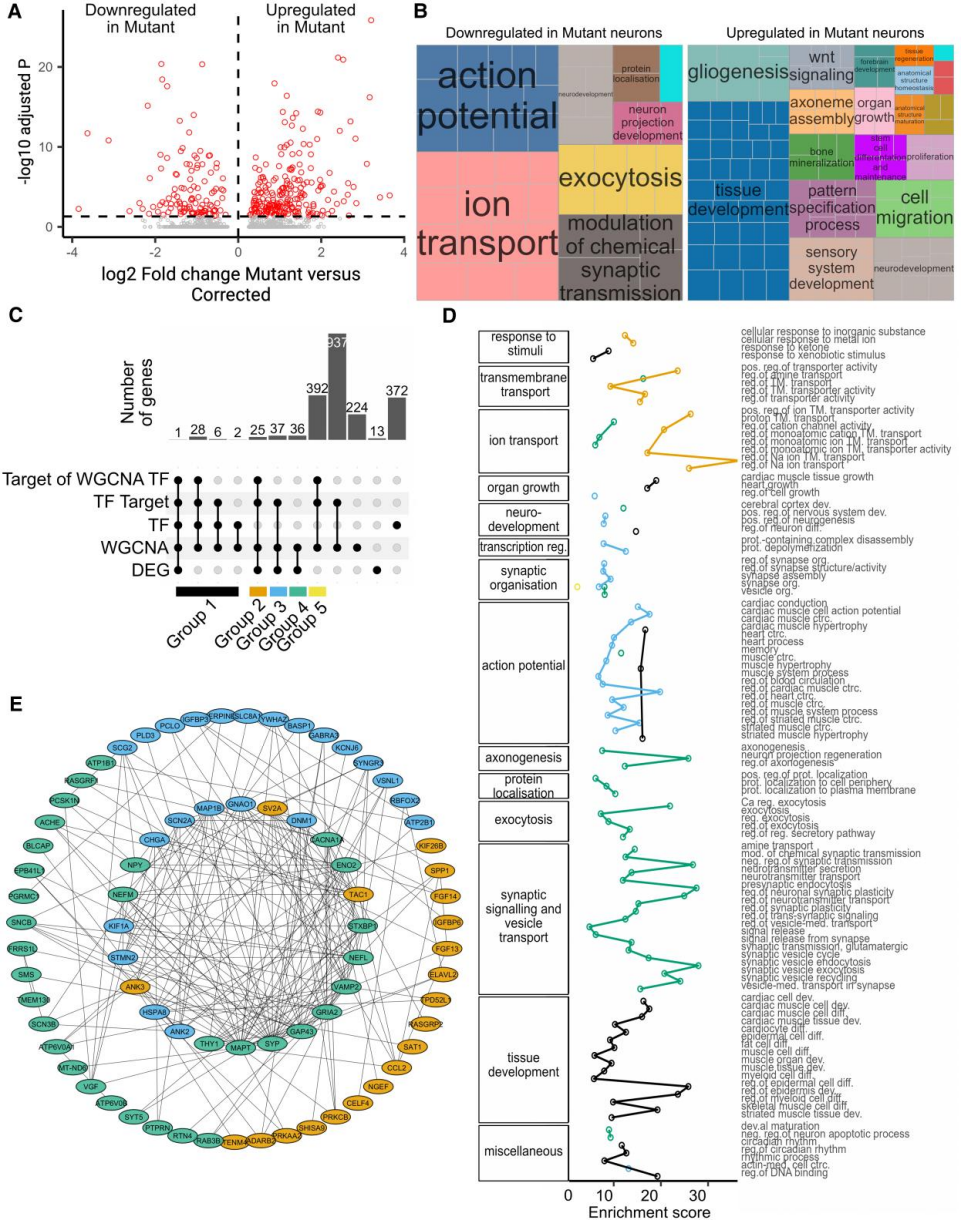
**Disrupted transcriptomic landscape of *SGCE*-mutation-positive medial ganglionic eminence-derived GABAergic inhibitory neurons.** (**A**) Volcano plot indicating the fold-change and adjusted *P*-values of differentially expressed genes. (**B**) Representative biological process gene ontology (GO) terms significantly over-represented in genes differentially up- and downregulated in *SGCE*-mutation-positive neurons. Similar GO terms are grouped by colour, based on semantic similarity and manual annotation. (**C**) Upset plot demonstrating the size of different gene groups categorized according to membership of differentially expressed genes, differentially enriched weighted gene co-expression network analysis (WGCNA) gene modules and SCENIC regulon analysis. (**D**) Biological process GO over-representation in five candidate gene groups (Groups 1–5). Similar GO terms are grouped together based on semantic similarity and manual annotation. Only GO terms significantly over-represented are shown (false discovery rate-adjusted *P*-value >0.05). (**E**) Protein–protein interaction network of genes in Groups 2–4. Ca = calcium-ion; ctrc. = contraction; DEG = differentially expressed genes; dev. = development; diff. = differentiation; med. = mediated; mod. = modulation; Mut = mutant; Na = sodium; neg. = negative; NPC = neural progenitors; org. = organization; pos. = positive; prot. = protein; reg. = regulated; reg.of = regulation of; RGL = radial glia; TF = transcription factors; TM. = transmembrane; WGCNA = weighted gene co-expression network analysis; WT = wild-type.

Differential gene expression analysis was undertaken of the cells in the Neuron1 and Neuron2 clusters to determine potential pathways impacted by *SGCE* mutation. Here, 222 and 112 genes met the threshold (adjusted *P*-value < 0.05 and log_2_ fold-change ≥ 0.25) as being significantly up- and downregulated, respectively, in the *SGCE*-mutation-positive neurons compared with their wild-type counterparts (Fig. 4A and Supplementary material, ‘Data 2 and 3’). GO term enrichment differed between these two groups of genes, with genes downregulated in mutant neurons enriched for GO terms related to action potential, ion transport, neuronal structure, synaptic signalling and organization, whereas genes upregulated in mutant neurons were enriched for terms related to tissue development, cell migration and response to stimuli (Fig. 4B and Supplementary material, ‘Data 4’).

To identify co-expressed genes correlated with the molecular mechanisms potentially underlying the transcriptomic changes observed in *SGCE*-mutation-positive neurons, weighted gene co-expression network analysis (WGCNA) was applied to determine associated gene modules. In total, 33 gene modules were identified, with modules M3, M10, M12, M13 and M18 being significantly negatively associated with and differentially enriched in the mutant genotype, whereas modules M16 and M25 were significantly positively associated with and differentially enriched in the mutant genotype (Supplementary Fig. 4A and B and Supplementary material, ‘Data 5’). Interestingly, almost 90% of the 112 differentially expressed genes downregulated in mutant neurons form part of the negatively associated and downregulated modules of mutant neurons, whereas <10% of the 222 upregulated differentially expressed genes were present in upregulated modules (Supplementary Fig. 4C), supporting the view that *SGCE* mutations lead primarily to transcriptional downregulation and loss of function.

To gain further insights into the changes underlying the downregulated genes in the *SGCE*-mutation-positive neurons, data derived from the differentially expressed genes, WGCNA and SCENIC regulon enrichment analyses were integrated. Here, 2073 genes were initially shortlisted from the union of: (i) WGCNA gene modules M3, M10, M12, M13 and M18; (ii) transcription factors of SCENIC regulons for which WGCNA gene modules were enriched; and (iii) differentially expressed genes downregulated in mutant neurons (Fig. 4C). Among these genes, five key groups emerged, including: transcription factors found in differentially enriched WGCNA modules (Group 1); differentially expressed WGCNA genes in regulons of Group 1 transcription factors (Group 2); differentially expressed WGCNA genes in other regulons (Group 3); differentially expressed WGCNA genes not in regulons (Group 4); and other WGCNA genes in regulons of Group 1 transcription factors (Group 5) (Fig. 4C and Supplementary material, ‘Data 6’). WGCNA analysis and protein–protein interaction analysis of these gene groups found that Groups 2–4 had the highest mean co-expression weight with genes within and across gene groups, and higher centrality measures in the protein–protein interaction network, but a significantly lower mean number of intragroup co-expression interactions than other groups (Supplementary Fig. 5A and B), suggesting that these gene groups are highly associated with the expression level of other genes and might be central to the transcriptomic landscape. GO enrichment analysis revealed that Groups 2–4 were significantly enriched for genes relating to distinct sets of GO terms, namely ion and transmembrane transport (Group 2), neurodevelopment, action potentials and synaptic organization (Group 3), and axongenesis, protein localization, exocytosis, and synaptic signalling and vesicle transport (Group 4). In contrast, Group 1 (all transcription factors) was predominantly enriched for GO terms related to action potential and tissue development (Fig. 4D and Supplementary material, ‘Data 7’).

These results suggest that *SGCE* mutations disrupt three facets of neuronal function: action potential generation; synaptic and vesicular transport; and axongenesis. To elucidate the relationship among Group 2–4 genes further, a protein–protein interaction network was constructed, based on identification of ‘hub’ genes with the highest measures of centrality measures in each group (Fig. 4E, Supplementary Fig. 5C and Supplementary material, ‘Data 8’). Group 4 genes demonstrated higher inter-group centrality measures (Supplementary Fig. 5C and Supplementary material, ‘Data 8’), suggesting that these genes (enriched for genes related to axongenesis, protein localization, exocytosis, synaptic signalling and vesicle transport) were likely to link Group 2 and Group 3 genes functionally. ‘Hub’ genes for inter-group interaction in Group 2–4 predominantly involved aspects of axonal organization and vesicular transport, for example: *GNAO1*, *MAP1B*, *STMN2* and *MAPT* (cytoskeletal structure and assembly); *DNM1* and *KIF1A* (transport machinery); *SV2A*, *SYP*, *VAMP2* and *STXBP1* (synaptic vesicle release); *TAC1*, *CHGA* and *NPY* (encoding precursors of vesicular transport); and *SCN2A*, *GRIA2* and *CACNA1A* (ion channels and receptors for membrane trafficking) (Fig. 4E). Interestingly, hub genes from Groups 2 and 3 also included *ANK2* and *ANK3*, respectively, encoding the protein ankyrin, central to the membrane localization and targeting of ion channels and neurotransmitter receptors.

### Disrupted neurite growth and arborization in *SGCE*-mutant GABAergic neurons

**Figure 5:**
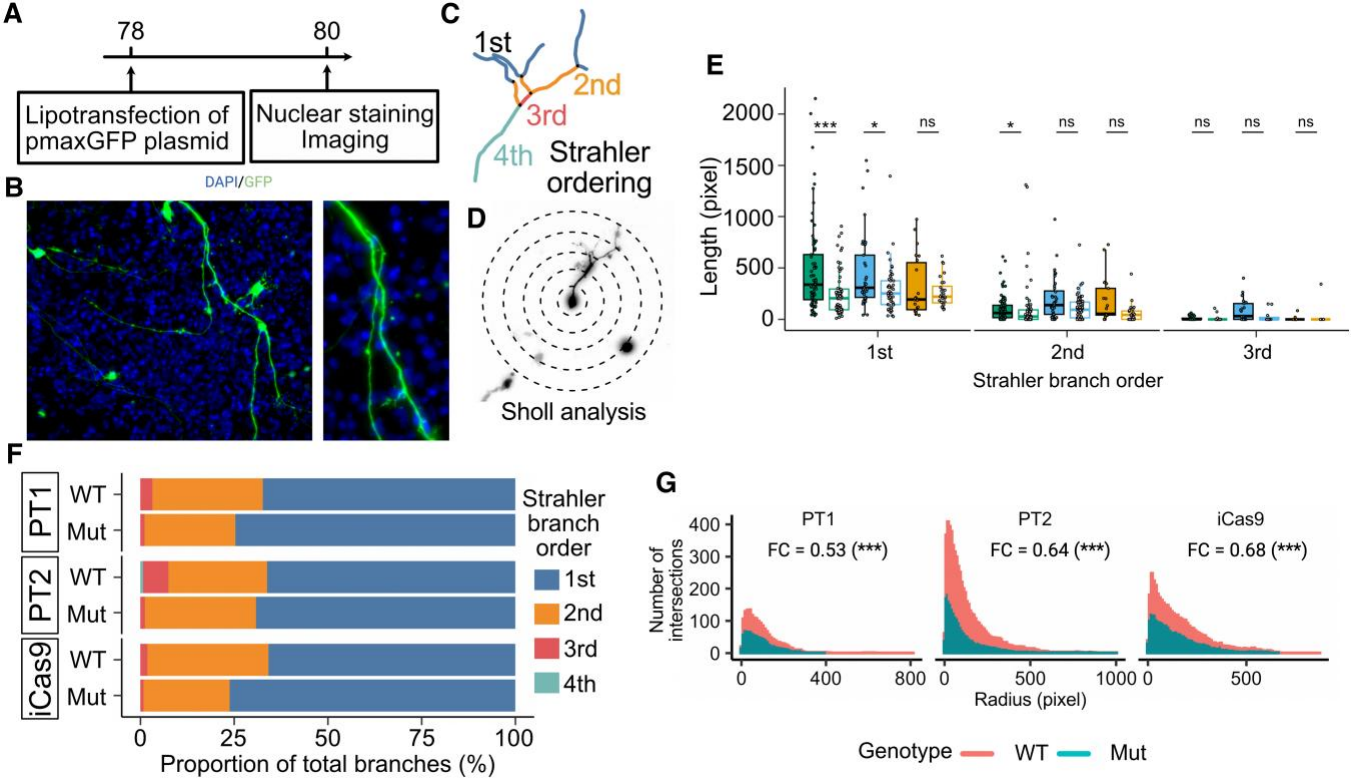
***SGCE* mutations disrupt dendritic architecture with reduced neurite outgrowth and branching.** (**A**) Schematic diagram of the experimental time line and (**B**) example images of neurite tracing. Magnified view shown on the *right*. Examples of Strahler branching analysis (**C**) and Sholl analysis (**D**) based on the neuron trace in **B**. (**E**) Box plot representation of the median and interquartile values of the length of Strahler order branches. Lines were compared using Wilcoxon signed-rank tests with false discovery rate correction. (**F**) Horizontal bar plot demonstrating the percentage proportion of each Strahler order branch type across each cell line. (**G**) Sholl analysis histogram of the number of dendritic intersections at sequential radial sizes (steps of 10-pixel size). Fold-change and significance symbols shown are the exponential of the coefficient and significance for genotype (mutant compared with wild-type) of Poisson regression models for each isogenic cell line pair. ns = not statistically significant (*P* > 0.05); *P < 0.05, ***P < 0.001. Number of cells analysed: iCas9wt = 24, iCas9ko = 36, Pt1 = 34, Pt1C = 28, Pt2 = 37, Pt2C = 32. FC = fold-change; Mut = mutant; PT1 = Patient 1; PT2 = Patient 2; WT = wild-type.

scRNAseq analysis implicated that *SGCE* mutations might lead to disrupted axonal organization and development, potentially manifested in the form of changes to dendritic architecture. Neurite length and branching complexity were examined in Day 80 neurons after sparse transfection with a constitutive enhanced green fluorescent protein (EGFP) expression plasmid (Fig. 5A and B and Supplementary Fig. 6) and analysed using the Strahler ordering system and Sholl analysis (Fig. 5C and D). Branching pattern analysis identified significantly shorter first-order branches [Fig. 5E; Patient 1 (PT1), *P* = 3.30 × 10^−4^; Patient 2 (PT2), *P* = 0.0477; iCas9, *P* = 0.798] but no significant differences in the length of higher-order branches (second and third) in *SGCE*-mutation-positive neurons compared with their isogenic wild-type controls (PT1, *P* = 3.30 × 10^−4^; PT2, *P* = 0.0477; iCas9, *P* = 0.798; Fig. 5E). In addition, fewer higher-order branches (second, third and fourth) were observed across the mutation-positive neurons compared with controls (PT1, *P* = 0.0362; PT2, *P* = 1.20 × 10^−3^; iCas9, *P* = 0.242; Fig. 5F). Sholl analysis was used to examine the branching complexity of the dendritic architecture, demonstrating that the presence of *SGCE* mutations showed a significant negative association with both neurite length and the number of branches across all three isogenically matched cell line pairs (PT1, *P* = 1.31 × 10^−61^; PT2, *P* = 4.11 × 10^−25^; iCas9, *P* = 9.73 × 10^−9^; Fig. 5G).

### Changes to intracellular calcium signalling response observed in *SGCE*-mutant GABAergic neurons

**Figure 6:**
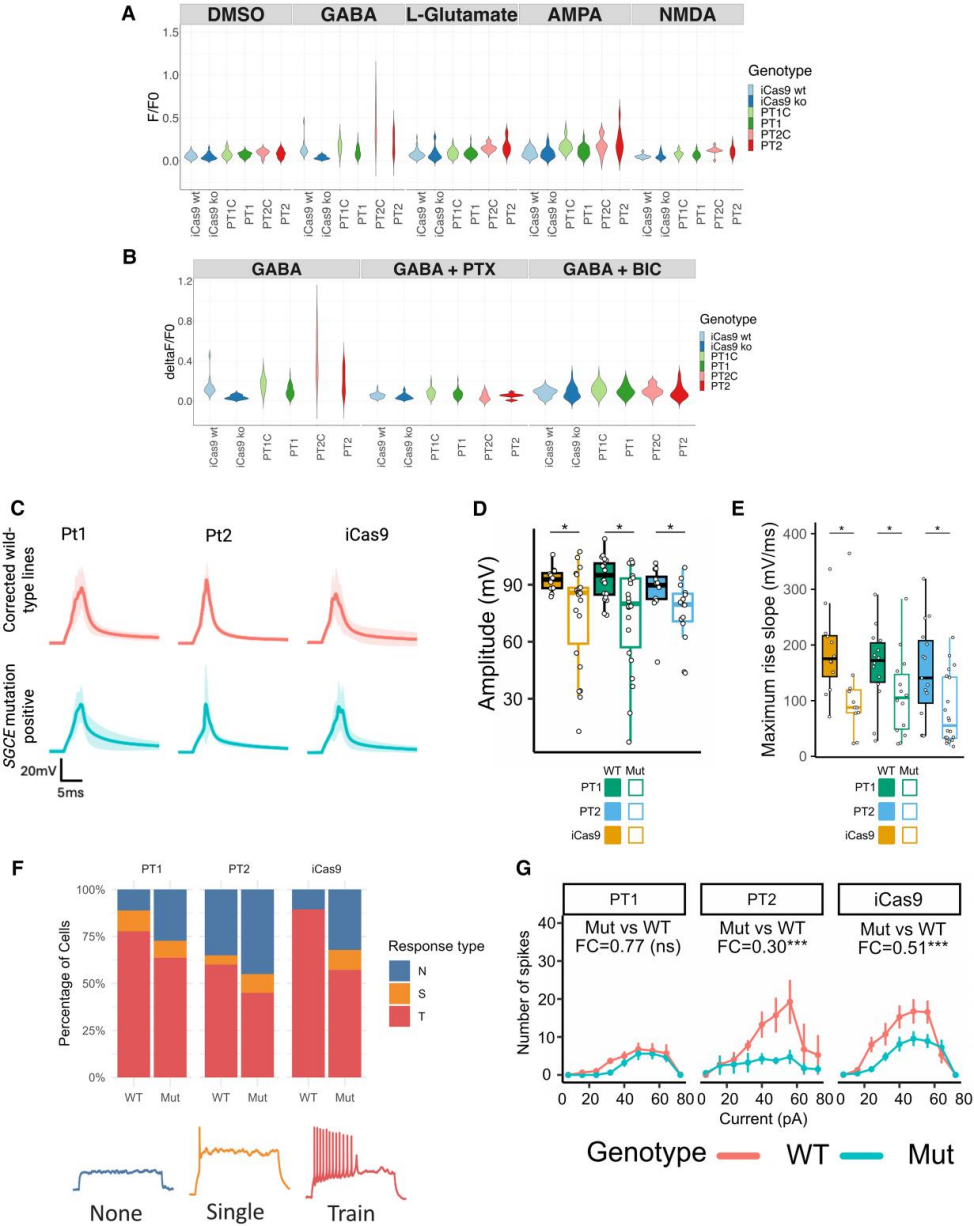
**Lower activity and excitability observed in *SGCE* mutation-positive medial ganglionic eminence-derived GABAergic neurons compared with wild-type controls.** (**A** and **B**) Violin plots comparing the peak calcium fluorescence response (Δ*F*/*F*_0_) following small molecule application. Lines were compared using one-way ANOVA (*n* = 15 wells per line per differentiation). Total recordings from 60 wells per line over three independent differentiations. (**C**) Average action potential trace evoked by 5 ms of 200 pA current stimulation. Shaded area represents the standard deviation of all cells. (**D** and **E**) Box plots representing median and interquartile range of the peak evoked amplitude (**D**) and maximum rise slope (**E**) of action potentials generated by short pulse electric stimulation. Lines compared using Wilcoxon signed-rank tests with false discovery rate correction. (**F**) Proportion of cells demonstrating no action potential (blue), single spikes (yellow) and train of spikes (red) following stimulation. (**G**) Average number of elicited action potential spikes. Error bars represent the standard error of means of all cells. Fold-change (FC) and significance symbols represent the exponential of the coefficient and significance for genotype (mutant compared with wild-type) in zero-inflated Poisson regression models of each isogenic cell line pair. ns = not significant; *P < 0.05, ***P < 0.001. Number of patch-clamp recordings: Pt1 = 14, Pt1C = 14, Pt2 = 20, Pt2C = 15, Pt9 = 12, Pt9C = 12. BIC = bicuculline; Mut = mutant; PT1 = Patient 1; PT2 = Patient 2; PTX = picrotoxin; WT = wild-type.

Although the changes to ion transport identified during RNA sequencing analysis might be broad, disruption to calcium signalling has been implicated across multiple monogenic forms of dystonia, including *SGCE*.^2,45^ As such a high-throughput calcium assay, using the FLIPR Penta system, was undertaken on Day 80 of differentiation. Compared with control dimethyl sulphoxide treatment conditions, overall intracellular peak response calcium concentrations were significantly higher in PSC-derived GABAergic neurons with application of GABA (*P* < 0.001), L-glutamate (*P* = 4.20 × 10^−4^) and AMPA (*P* < 0.001) but not NMDA (*P* = 0.982) (Fig. 6A). Independent co-application of the GABA and GABA_A_R antagonists bicuculline (*P* < 0.001) and picrotoxin (*P* < 0.001) led to a significant reduction in the GABA-induced calcium response across all cell lines (Fig. 6B). The same significant reduction was also observed with co-application of CNQX (AMPA-R antagonist) and L-glutamate (*P* = 0.012), and CNQX and AMPA (*P* < 0.001). In contrast, application of AP5 (NMDA-R antagonist) with L-glutamate identified no significant reduction (*P* = 0.18) (Supplementary Fig. 7A). Representative calcium traces are shown in Supplementary Fig. 7B and C. Significant differences between *SGCE*-mutation-positive and wild-type lines were observed only with the GABA-induced response, with significantly larger amplitudes observed in those with wild-type sequence across all three cell line pairs (iCas9, *P* = 0.03; PT1, *P* = 0.02; PT2, *P* < 0.001; Fig. 6B). No significant differences were observed across the cell line pairs in their response to either GABA_A_R antagonist (Fig. 6B), potentially indicating that this effect is mediated by a difference in GABA_A_R and supported by the finding of fewer and smaller spontaneous postsynaptic currents outlined below. It should also be noted that the calcium measures undertaken in this study were made at a network level from individual well cultures, and therefore likely to be included with these recordings is a signal generated from cell types other than MGE-derived GABAergic neurons.

### Reduced activity and excitability in *SGCE*-mutation-positive GABAergic neurons

Transcriptomic analysis additionally suggested *SGCE* mutations to be associated with disruption of action potential generation and synaptic transmission. To explore potential changes further, whole-cell patch-clamp analysis was initially undertaken across current- and voltage-clamp modes for all cell lines. No significant differences in passive membrane properties were observed between each *SGCE*-mutation-carrying line and their isogenic control (Supplementary Fig. 8). However, in response to short-pulse electrical stimulation, action potentials generated from *SGCE*-mutation-carrying neurons were of significantly smaller amplitude (PT1, *P* = 0.0225; PT2, *P* = 0.0281; iCas9, *P* = 0.0281) and had a lower maximum rise slope (PT1, *P* = 0.027; PT2, *P* = 0.0165; Pt9, *P* = 0.0165) compared with their wild-type counterparts (Fig. 6C–E), whereas no significant differences were observed across the other measured kinetic metrics (Supplementary Fig. 8). With sustained electrical stimulation, fewer *SGCE*-mutation-positive neurons exhibited a repetitive firing pattern compared with their isogenic control (Fig. 6F), and significantly fewer action potential spikes were observed with the mutant genotype following sustained electric stimulation (PT1, *P* = 0.253; PT2, *P* = 2.80 × 10^−6^; iCas9, *P* = 2.55 × 10^−6^; Fig. 6G).

**Figure 7:**
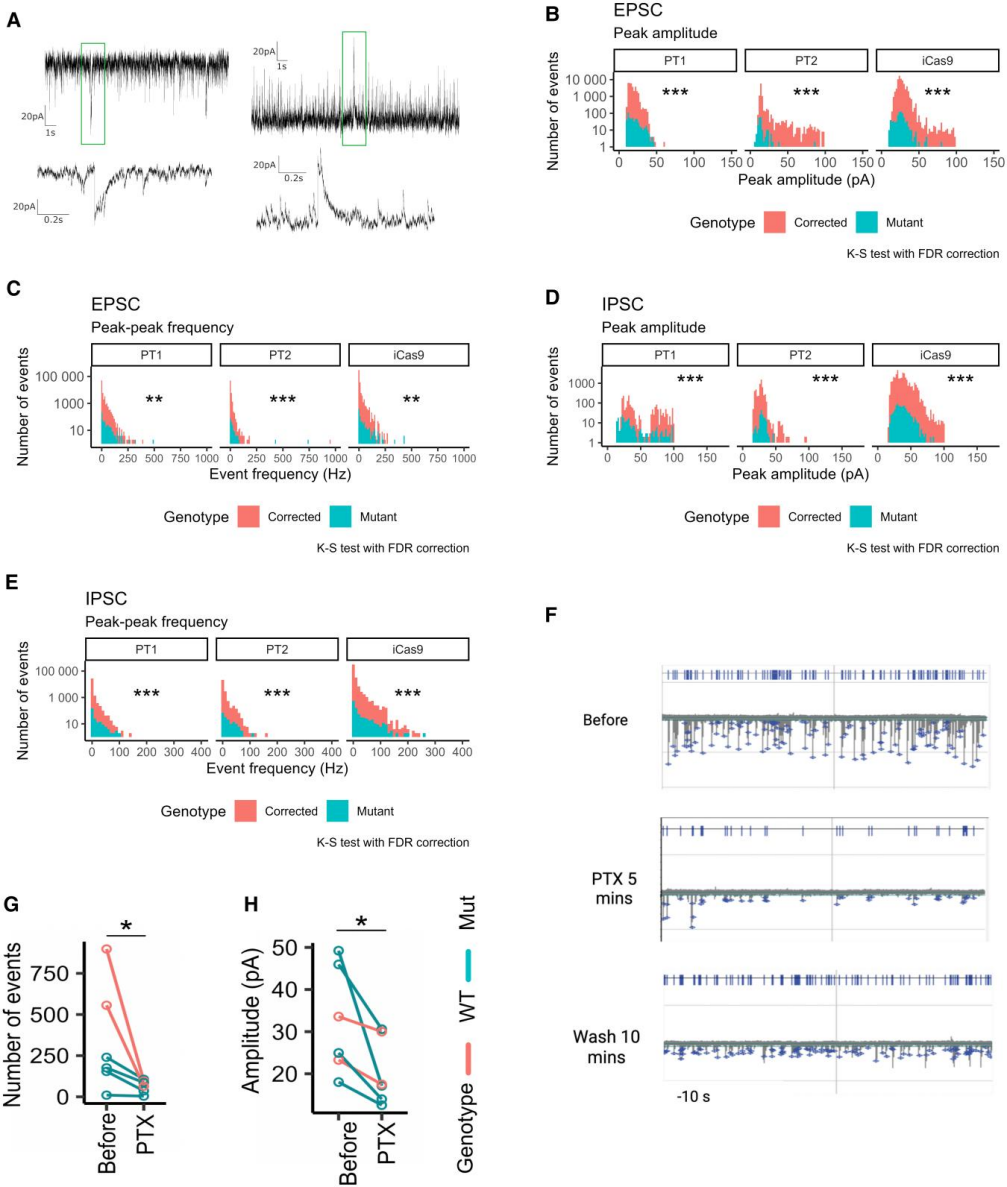
**Reduced spontaneous excitatory and inhibitory currents in *SGCE* mutation-positive medial ganglionic eminence-derived GABAergic neurons compared to wild-type controls.** (**A**) Example traces of sEPSCs and sIPSCs. (**B** and **C**) The amplitude (**B**) and frequency (**C**) of sEPSC events over 3 min recordings. (**D** and **E**) The amplitude (**D**) and frequency (**E**) of sIPSC events over 3 min recordings. Two-sample Kolmogorov–Smirnov (K-S) test used to compare the distribution of postsynaptic current amplitudes between mutant and wild-type neurons (number of recordings: Pt1 = 32, Pt1C = 41, Pt2 = 22, Pt2C = 16, Pt9 = 27, Pt9C = 27). (**F**) Example trace of spontaneous postsynaptic current with cells held at −60 mV at baseline (prior), following picrotoxin (PTX) application and following a subsequent wash. (**G** and **H**) Number (**G**) and median amplitude (**H**) of postsynaptic current events over 3 min recordings before and during PTX application. Pre- and post-treatment were compared using Wilcoxon signed-rank tests with false discovery rate correction (number of recordings: total = 6, *SGCE*-mutation-positive = 4, wild-type control = 2). ns = not significant; *P < 0.05, **P < 0.01, ***P < 0.001. BIC = bicuculline; FDR = false discovery rate; Mut = mutant; PT1 = Patient 1; PT2 = Patient 2; sEPSC = spontaneous excitatory postsynaptic current; sIPSC = spontaneous inhibitory postsynaptic current; WT = wild-type.

Spontaneous postsynaptic excitatory and inhibitory currents were recorded by holding the membrane potential at −60 and 0 mV, respectively (Fig. 7A). Here, both spontaneous excitatory postsynaptic currents (sEPSCs) and spontaneous inhibitory postsynaptic currents (sIPSCs) were observed to be of lower amplitude [sEPSC: Patient 1 (Pt1) = 3.39 × 10^−10^, Patient 2 (Pt2) < 0.001, iCas9 < 0.001; sIPSC: Patient 1 (Pt1) < 0.001, Patient 2 (Pt2) < 0.001, iCas9 < 0.001] and lower frequency [sEPSC: Patient 1 (Pt1) = 5.43 × 10^−8^, Patient 2 (Pt2) = 4.22 × 10^−3^, iCas9 = 8.59 × 10^−3^; sIPSC: Patient 1 (Pt1) = 1.07 × 10^−4^, Patient (Pt2) = 8.89 × 10^−6^, iCas9 = 2.40 × 10^−14^] in *SGCE*-mutation-positive neurons compared with their isogenic wild-type controls (Fig. 7B–E). Furthermore, the sEPSC are likely to be dependent on GABA_A_R signalling, given the reduction in both frequency and amplitude with the application of the GABA_A_R antagonist, picrotoxin (Fig. 7F–H).

**Figure 8:**
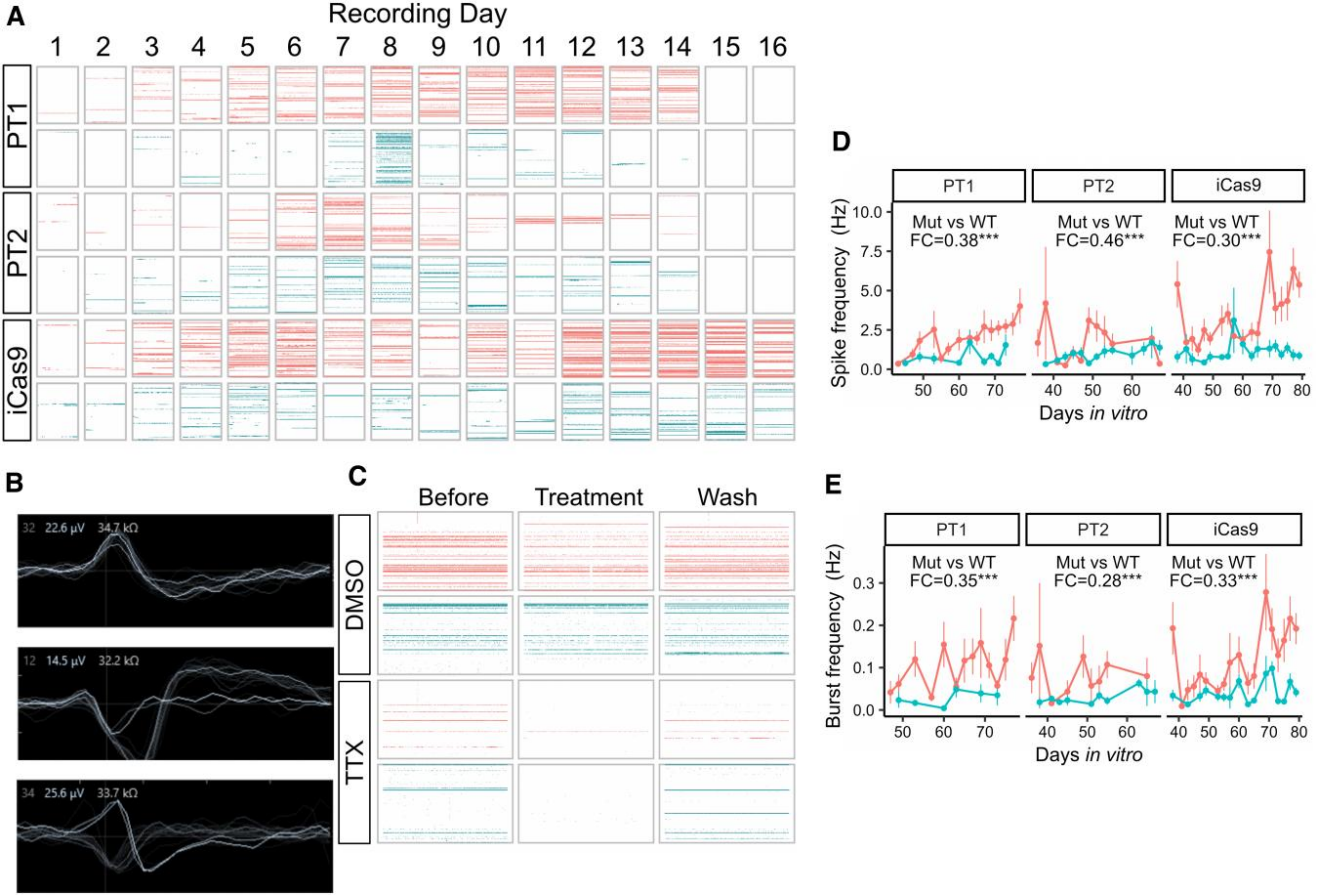
**Reduced network level neuronal activity in *SGCE*-mutation-harbouring lines.** (**A** and **B**) Raster plot (**A**) and representative waveforms (**B**) of neuronal activity captured using MEAs. The raster plot (**A**) demonstrates average spike frequency across each of the 16 recording electrodes (represented as rows of horizontal dots per electrode when activity was detected), with cell lines indicated on the *y*-axis and day or recording on the *x*-axis. (**C**) Raster plot of neuronal activity before, during tetrodotoxin treatment and following a subsequent wash. (**D**) MEA captured spike frequency across all cell line pairs. (**E**) MEA captured burst frequency across all cell line pairs. Data are presented as line plots, with each point representing the mean value across a minimum of three wells per experiment, from three independent experiments per line. Fold-change (FC) and significance symbol shown are the exponential of the coefficient and significance for genotype (mutant compared with wild-type) in gamma regression models for each isogenic cell line pair. ns = not significant; ***P < 0.001. Recordings were captured from 12 wells per cell line, per differentiation, resulting in recordings from 36 wells per cell line. MEA = multi-electrode array; Mut = mutant; TTX = tetrodotoxin; WT = wild-type.

Use of MEAs to evaluate network-level neuronal activity demonstrated an overall increase in activity over time, across all cell lines, indicating progressive development of neuronal functional maturity (Fig. 8A and B). The MEA-detected activity was reduced across both mutant and wild-type lines with application of the voltage-gated sodium channel blocker, tetrodotoxin, supporting the biological relevance of the MEA-detected activity, given its dependence on voltage-gated sodium channels, a feature of neuroectodermally derived differentiated neurons (Fig. 8C).^46^  *SGCE*-mutation-positive neurons again demonstrated lower levels of activity, with significantly lower spike frequency (PT1, *P* = 1.82 × 10^−6^; PT2, *P* = 8.64 × 10^−11^; iCas9, *P* = 4.62 × 10^−20^; Fig. 8D) and burst frequency (PT1, *P* = 2.57 × 10^−4^; PT2, *P* = 4.55 × 10^−13^; iCas9, *P* = 4.73 × 10^−9^; Fig. 8E) compared with their isogenic wild-type controls. Collectively, *SGCE*-mutation-positive GABAergic neurons were less active and excitable and demonstrated fewer spontaneous postsynaptic currents and lower spike and burst network activity, in comparison to each of their wild-type controls.

## Discussion

Here, we have demonstrated successful differentiation of MGE-patterned GABAergic neurons derived from two patient-derived *SGCE*-mutation-positive iPSC lines and a single *SGCE* knockout human ESC line, coupled with their isogenic wild-type control lines. The generated neurons resemble human fetal MGE-derived GABAergic neurons when compared with previously reported datasets, in terms of both developmental markers and maturity. Subsequent single-cell transcriptomic analysis identified disruption to axonal and cytoskeletal organization, vesicular transport, synaptic signalling and action potential generation in the *SGCE*-mutation harbouring lines compared with controls. Further support for these differences was observed across multiple functional assays, including reduced neurite outgrowth and branching complexity, lower intracellular calcium response to GABA stimulation, and a reduction in the neuronal network activity and excitability in the *SGCE*-mutant GABAergic neurons compared with their wild-type controls. These phenotypes are in contrast to the hyperexcitable phenotype observed in *SGCE*-mutant cortical glutamatergic neurons and are consistent with the loss-of-inhibition hypothesis widely reported in dystonia pathogenesis.^47^

Dystonia is widely considered to be a network disorder, caused by disruption to neuronal activity rather than by the loss of specific neuronal subtypes. Consistent with the absence of gross structural abnormalities in MRI studies, we identified no significant differences in the expression of key developmental markers for MGE-derived GABAergic neurons between mutant and control cell lines, in keeping with previous *SGCE*-mutation-positive stem cell models differentiated towards cortical glutamatergic neurons and striatal medium spiny neurons.^2^ In addition, scRNAseq analysis of the MGE-derived GABAergic neurons derived in this study found them to resemble their human fetal equivalents and to exhibit a similar transcriptomic maturity to PSC-derived MGE-patterned GABAergic neurons from other studies, supporting the authenticity of the model derived.^44^ However, it should be noted that PSC-derived MGE-patterned GABAergic neurons are likely to be a mixed population of cortical GABAergic interneurons (LHX6, MAF, MAFB and ERBB4), globus pallidus GABAergic projection neurons (LHX6, NKX2.1 and LHX8) and striatal GABAergic interneurons (LHX6, NKX2.1). In addition, differences in differentiation efficiency were observed between results obtained from immunohistochemistry of key MGE-patterned GABAergic neurons and scRNAseq, probably owing to variations in differentiation efficiency across batches. Previous studies have implicated a role for both cortical GABAergic interneurons and globus pallidus projection neurons in the pathogenesis of dystonia, with transcranial magnetic stimulation reducing intracortical inhibition in the primary motor cortex,^48^ whereas intraoperative recordings have demonstrated reduced and irregular firing and burst activities^49^ and excessive synchronized oscillation in the globus pallidus,^50^ while deep brain stimulation of the globus pallidus internus has proved to be an effective treatment for dystonia.^51^

Detailed scRNAseq comparison between *SGCE*-mutant and wild-type neurons identified a network of downregulated genes affecting specific aspects of neuronal function, including axonal and cytoskeletal organization, vesicular transport, synaptic signalling and action potential generation. More specifically, significantly downregulated genes have been linked with a number of these functions, including cytoskeletal organization (*MAP1B*, *STMN2* and *MAPT*), membrane localization (*EPB41LI* and *TENM4*) and anchoring of ion channels and receptors (*ANK2* and *ANK3*), supporting the previously hypothesized role for ε-sarcoglycan in these processes.^52^ Other highlighted genes have been linked with synaptic function, including vesicle release (*VAMP2*, *SV2A* and *SYP*),^53,54^ and ion channel gating and action potential generation (*SCN2A*, *CACNA1A* and *SLC8A1*).^55,56^ In addition, several of these downregulated genes have been implicated in clinical disorders linked with neurological and neuropsychiatric phenotypes in which disruption to the balance between excitatory and inhibitory neuronal activity is considered to be of importance, including dystonia, with examples of such genes being *GNAO1*, *STXBP1* and *KIF1A*.^57-59^ Furthermore, the discovery of dystonia-related genes as being dysregulated hubs in *SGCE*-mutant GABAergic neurons implicates a potential convergence of the molecular and cellular mechanisms underlying dystonia, which could be explored further using iPSCs of other monogenic forms of dystonia.

The subsequent structural and functional assays undertaken in this study support multiple findings from the transcriptomic analysis, including disruption to the dendritic architecture with shorter branches, fewer higher-order branches and a less complex branching morphology in *SGCE*-mutation-positive lines compared with their isogenic controls. Multiple factors are recognized to impact dendritic morphology, including actin cytoskeletal regulators, neuronal activity, membrane receptors and disruption to calcium signalling,^60-62^ with the lower calcium signalling response following application of GABA, coupled with reduced single-cell and network functional activity in *SGCE*-mutation-positive neurons, in comparison to their wild-type counterparts observed in this study, potentially contributing to this change in morphology. However, as outlined previously, these relationships are likely to be bidirectional, with evidence supporting that changes to dendritic complexity have a secondary impact on neuronal activity and firing patterns.^63^

The multiple functional assays undertaken in this study likewise demonstrated *SGCE*-mutation-positive MGE-derived interneurons to be less active, with fewer action potential spikes and postsynaptic currents at a single-cell level, whereas network-level MEA analysis demonstrated lower spike and burst frequency compared with their wild-type isogenic controls. Our transcriptomic analyses provide potential indications to the factors that might be contributing to these changes, including downregulation of genes involved in the generation of voltage-gated sodium channels (*SCN2A* and *SCN3B*), AMPA receptor components (*GRIA2* and *FRRS1L*) and inwardly rectifying potassium channels (*KCNJ6*), avenues for potential exploration in future work. Evidence increasingly indicates that dystonia arises from disruption to neuronal networks, shaped by an imbalance between excitatory and inhibitory signalling centred around a loss of inhibition.^64,65^ Our previous work has demonstrated that patient-derived *SGCE*-mutation-carrying iPSC lines differentiated towards an excitatory glutamatergic lineage harbour a hyperexcitable phenotype, whereas the present study implies that, at a cortical level, this is accompanied by a reduction in inhibitory interneuronal activity.^2^ Given that both these studies have involved two-dimensional monocultures of each individual cell line, future work will require co-culture of both neuronal subtypes in order to determine ongoing existence of these phenotypes in the company of the other cell type, and whether their effects are additive or whether one predominates in a co-culture system.^66^

## Conclusion

In conclusion, this study demonstrates reduced excitability in MGE-derived GABAergic neurons harbouring *SGCE* mutations, potentially additive to the observed hyperexcitability in *SGCE*-mutant cortical glutamatergic neurons, contributing to the hyperkinetic phenotype of myoclonus dystonia. Underlying the electrophysiological defects, scRNAseq revealed dysregulated gene networks centred on specific neuronal functions, including cytoskeletal organization, anterograde trafficking and membrane localization of ion channels and neurotransmitter receptors, consistent with the potential biological function of ε-sarcoglycan in cytoskeletal organization and membrane localization. Future work will involve a direct assessment of how *SGCE* mutations impact the cortico-basal ganglia–thalamo-cortical neuronal network and the molecular mechanisms by which *SGCE* mutations affect network excitability in different neuronal subtypes.

## Supplementary Material

awaf272_Supplementary_Data

## Data Availability

The sequencing data discussed in this publication have been deposited in NCBI's Gene Expression Omnibus and are accessible through accession number GSE280716. Scripts used for data analyses are available on GitHub, accessible via https://github.com/leabram/InterneuronBrain2025. Raw data are available upon reasonable request.
