# Supplementary material for: Transcriptomic disruption and hypoactivity in DYT-SGCE medial ganglionic eminence-patterned inhibitory neurons
Source: Brain. 2025 Jul 25;149(1):209–25. doi: 10.1093/brain/awaf272 (PMC12782168; doi:10.1093/brain/awaf272)
Supplement: awaf272_Supplementary_Data [file awaf272_supplementary_data.zip › brain-2025-00169-File010.pdf]

Supplementary Material

Supplementary Figure 1: Canonical marker expression of medial ganglionic eminence-derived GABAergic neuron differentiation.

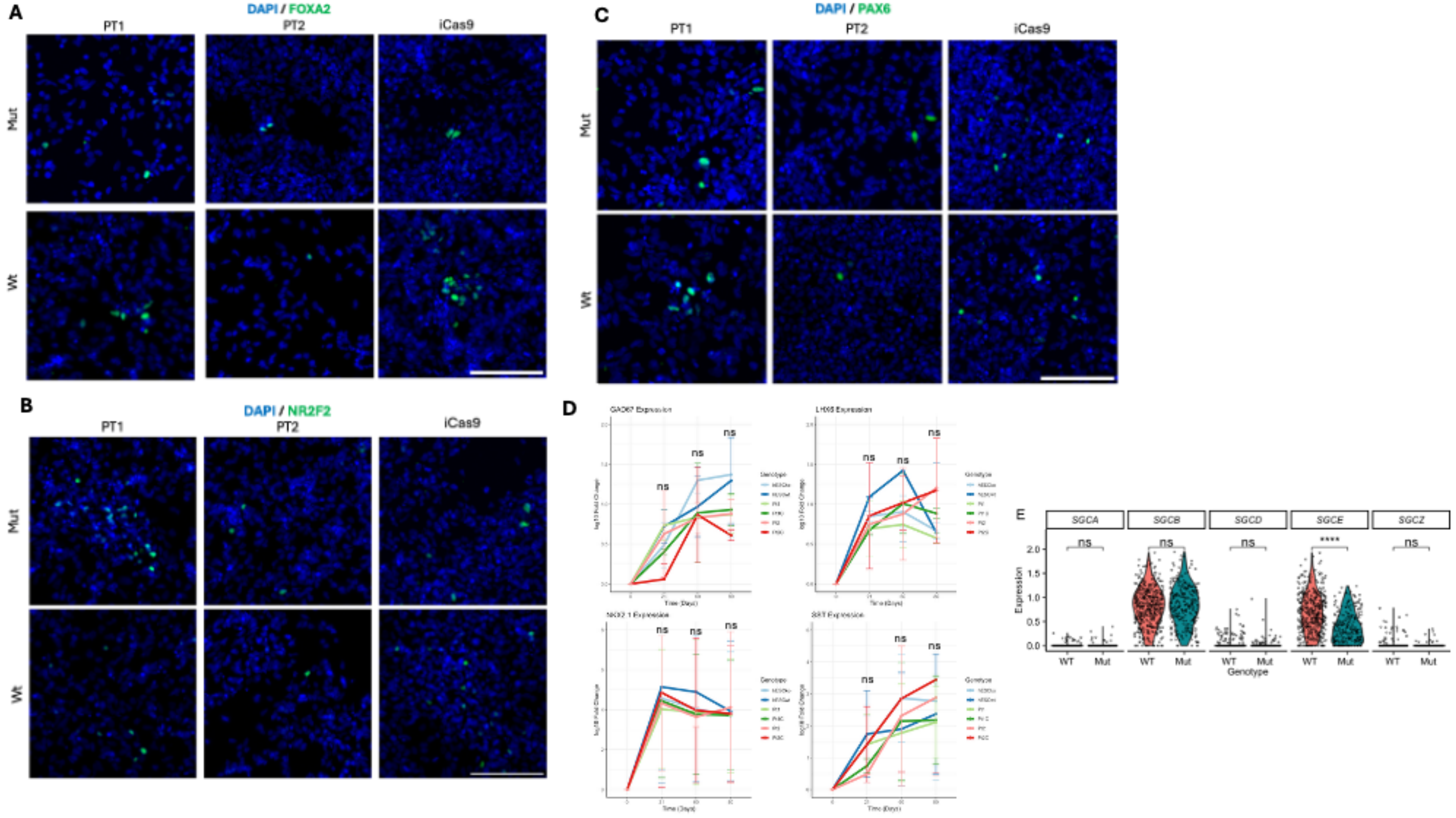

Legend: Representative immunofluorescence images of the expression of FOXA2 (ventral midbrain marker) (**A**), NR2F2 (caudal ganglionic eminence marker) (**B**), and PAX6 (lateral ganglionic eminence and dorsal forebrain marker) (**C**) on day 25 differentiation. Scale bar = 100 µm. **D:** qRT-PCR expression at Days 0, 21, 60 and 80 for markers of MGE-derived GABAergic neuronal markers, including *GAD67*, *LHX6*, *NKX2.1* and *SST*. Each SGCE mutation carrying line is compared to their wild-type isogenic control. Data presented as mean±SEM from 3 independent experiments per line. Lines compared using two-way ANOVA analysis. **E:** Violin plot showing the normalised expression of genes encodings proteins of the sarcoglycan family in single-cell RNA-sequencing. Lines compared using Wilcoxon signed-rank tests with FDR correction. Mut: mutant; WT: wild-type. *p<0.05. **p<0.01, ***p<0.001, ns: not significant.

Supplementary Figure 2: Processing and annotation of single-cell RNA sequencing data.

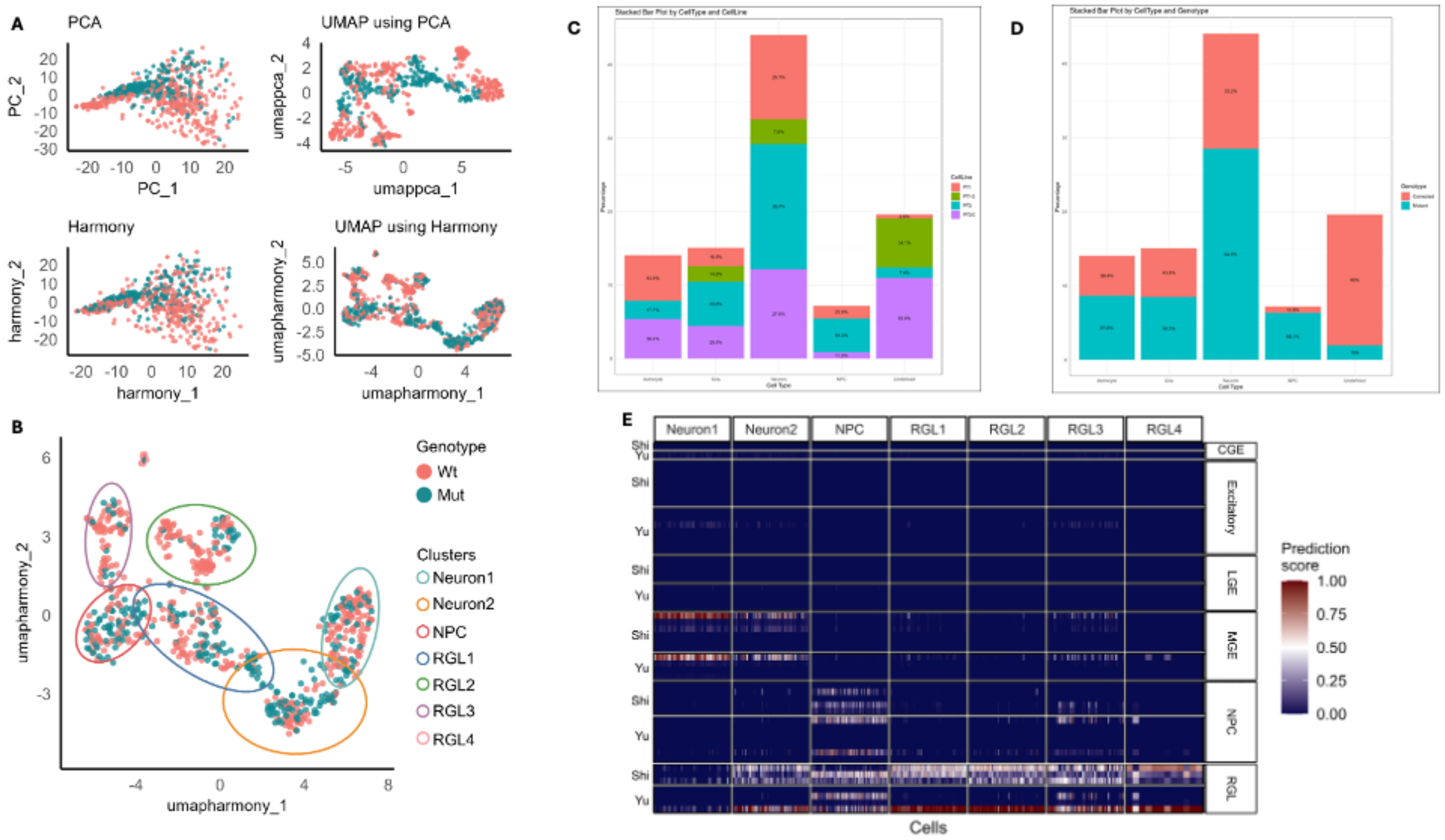

Legend: A: Harmony integration aligned wild-type (WT) and mutant (Mutant) cells on Uniform Manifold Approximation and Projection (UMAP) plot coloured by genotype. **B:** D80 UMAP plot of single-cell RNA-sequencing (scRNAseq) of WT and Mut medial ganglionic eminence (MGE)-derived GABAergic neurons. Coloured by genotype and each cluster identified by distinct circumferential colour. C: Stacked bar plot demonstrating the proportion of profiled cells by cell type. D: Stacked bar plot demonstrating the proportion of *SGCE* mutant and isogenic control profiled cells per cell type. **E:** Heatmap of prediction score of reference mapping to human foetal brain datasets (Shi: PMID 34737447; Yu: PMID 34882453). CGE: caudal ganglionic eminence; LGE: lateral ganglionic eminence; MGE: medial ganglionic eminence; Mut: mutant; NPC: neural progenitor; PCA: principal component analysis; RGL: radial glia; UMAP: uniform manifold approximation and projection; WT: wild-type.

Supplementary Figure 3: Integrated transcriptomic analysis of pluripotent stem cell-derived medial ganglionic eminence-lineage GABAergic neurons.

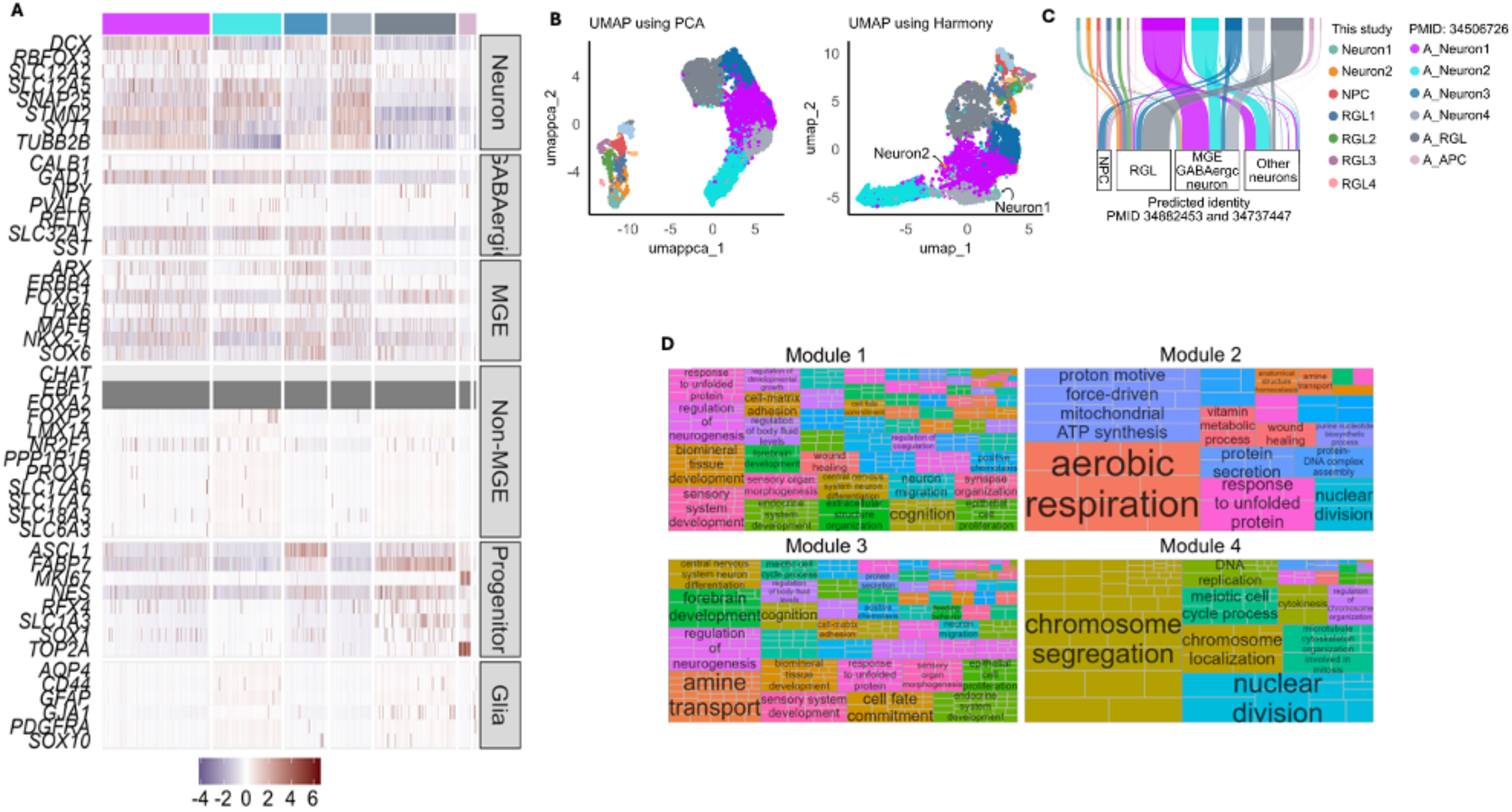

Legend: **A:** Heatmap of the expression of canonical marker genes for distinct lineages and cell types in differing clusters of the Allison *et al.* dataset. **B** UMAP plot before and after Harmony integration of this study and Allison *et al.* dataset (PMID 34506726). **C** Sankey plot demonstrating the predicted identity of the integrated pluripotent stem cell (PSC)-derived dataset based on two published human foetal MGE scRNAseq datasets. **D:** Averaged gene ontology enrichment of gene modules dynamically regulated along the pseudo-temporal trajectory. Similar GO terms are grouped with the same colour based on semantic similarity and manual annotation. **Key:** APC: astrocyte progenitor cell; MGE: medial ganglionic eminence; NPC: neural progenitor; RGL: radial glia; UMAP: uniform manifold approximation and projection.

Supplementary Figure 4: Single-cell RNA sequencing comparison of wild-type and *SGCE*-mutation positive medial ganglionic eminence-derived GABAergic neurons.

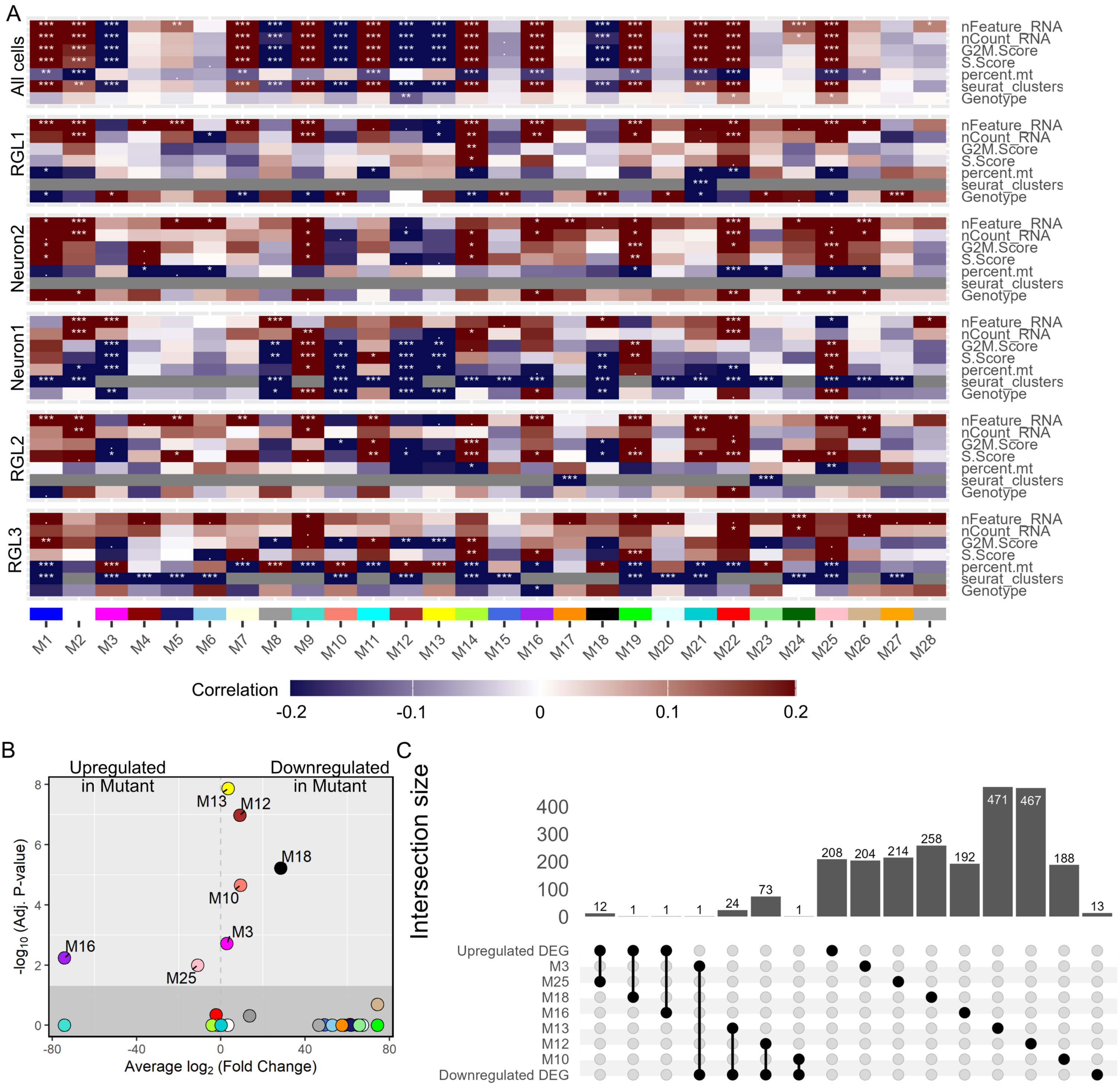

Legend: **A:** Heatmap reflecting the correlation between weighted gene co-expression network analysis (WGCNA) gene modules and different trait variables. **B:** Volcano plot representation of differentially expressed module eigengene analysis, reflecting up- and down-regulated gene modules s individual circles. **C:** Upset plot demonstrating the overlap between differentially expressed gene modules and differentially expressed genes. Key: DEG: differentially expressed genes; Mut: mutant; NPC: neural progenitor; RGL: radial glia; WT: wild type.

Supplementary Figure 5: Network analysis of candidate gene groups underlying the changes in transcriptomic landscape of *SGCE*-mutation positive neurons compared to wild-type controls.

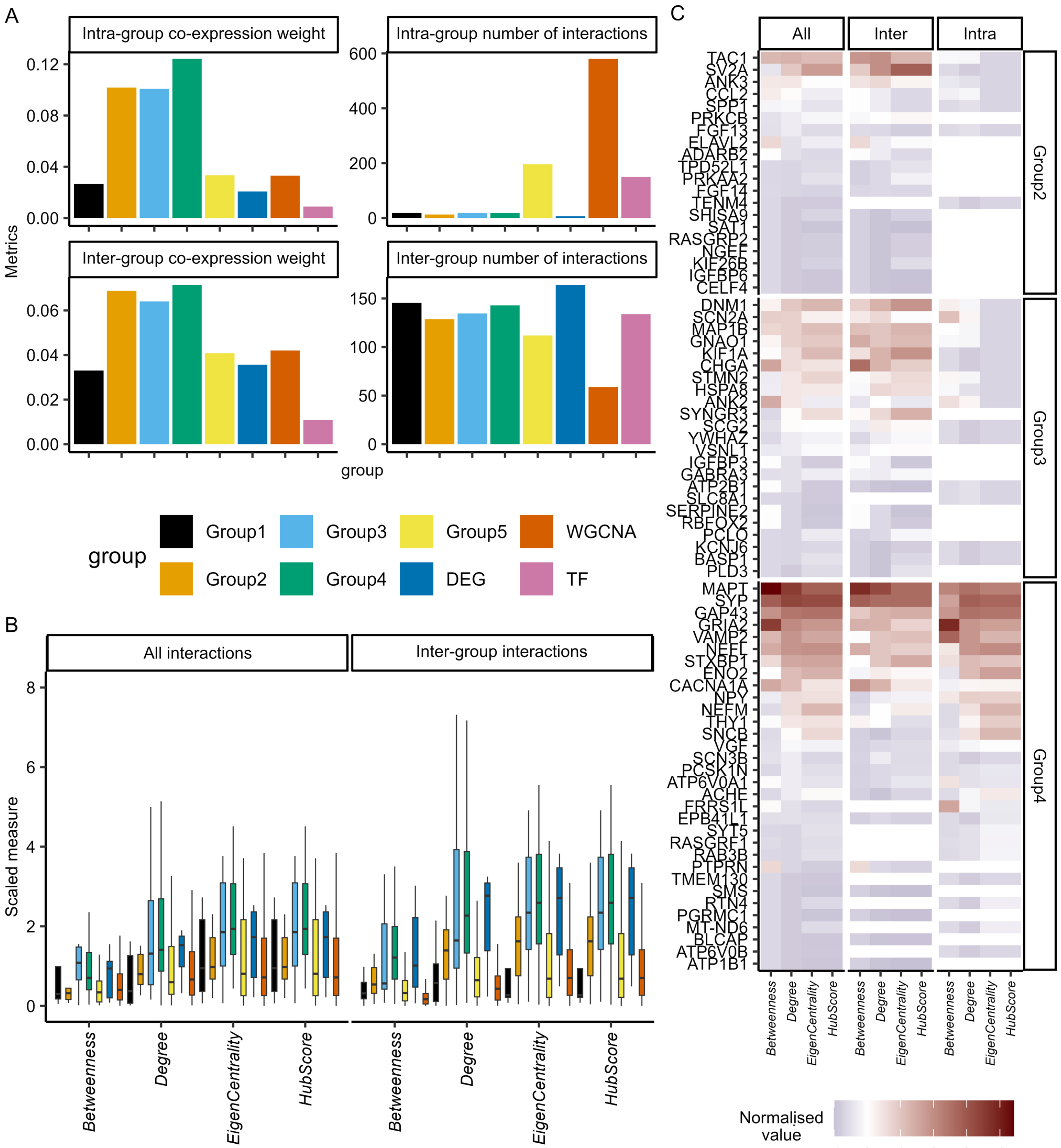

Legend: **A:** Bar plots representing the co-expression weight and number of co-expression edges of the weighted gene co-expression network analysis (WGCNA) across gene groups 1-5, coupled with the DEG, WGCNA and transcription factor groups. **B:** Bar plot comparing network analysis metrics of the protein-protein interaction network across the same distinct gene groups. **C:** Heatmap demonstrating different centrality measures of protein-protein interaction network analysis of Group 2-4 genes. Key: DEG: differentially expressed genes; Inter: inter-group; Intra: intra-group TF: transcription factor; WGCNA: weighted gene co-expression network analysis

Supplementary Figure 6: Immunohistochemical images demonstrating dendritic branching at D80 differentiation

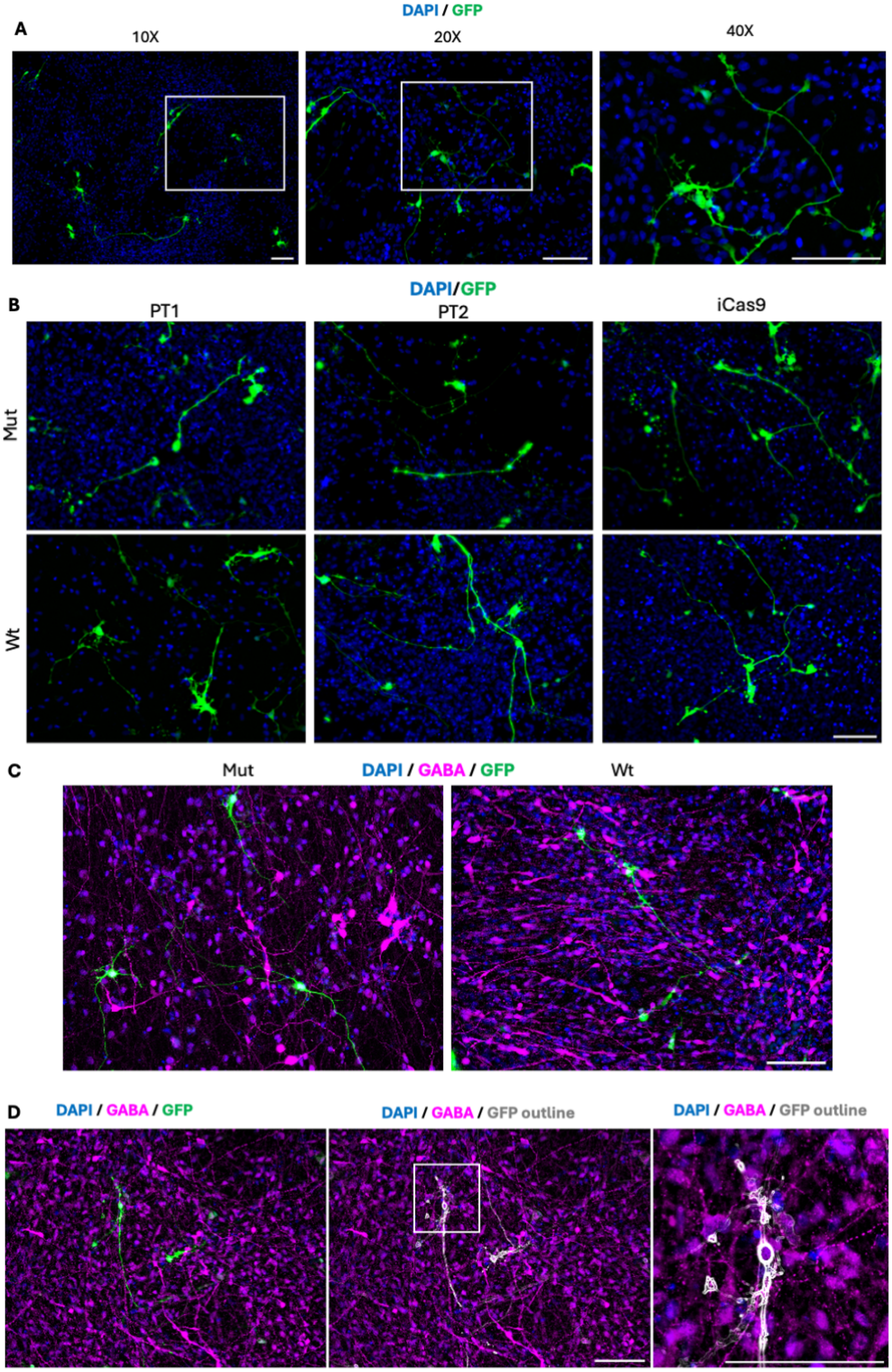

Legend: A-B: Representative immunofluorescence images of GFP-labelled neurites across x10, x20 and x40 magnification (**A**) and for all cell lines at x20 magnification (**B**). **C-D**: Representative immunofluorescence images demonstrating GFP-labelled neurites co-stained with MGE-derived interneuron marker GABA (**C**) with magnified example of double staining (**D**). Scale bar = 100µm.

Supplementary Figure 7: Comparison of calcium signalling in wild-type and *SGCE*-mutation positive medial ganglionic eminence-derived GABAergic neurons.

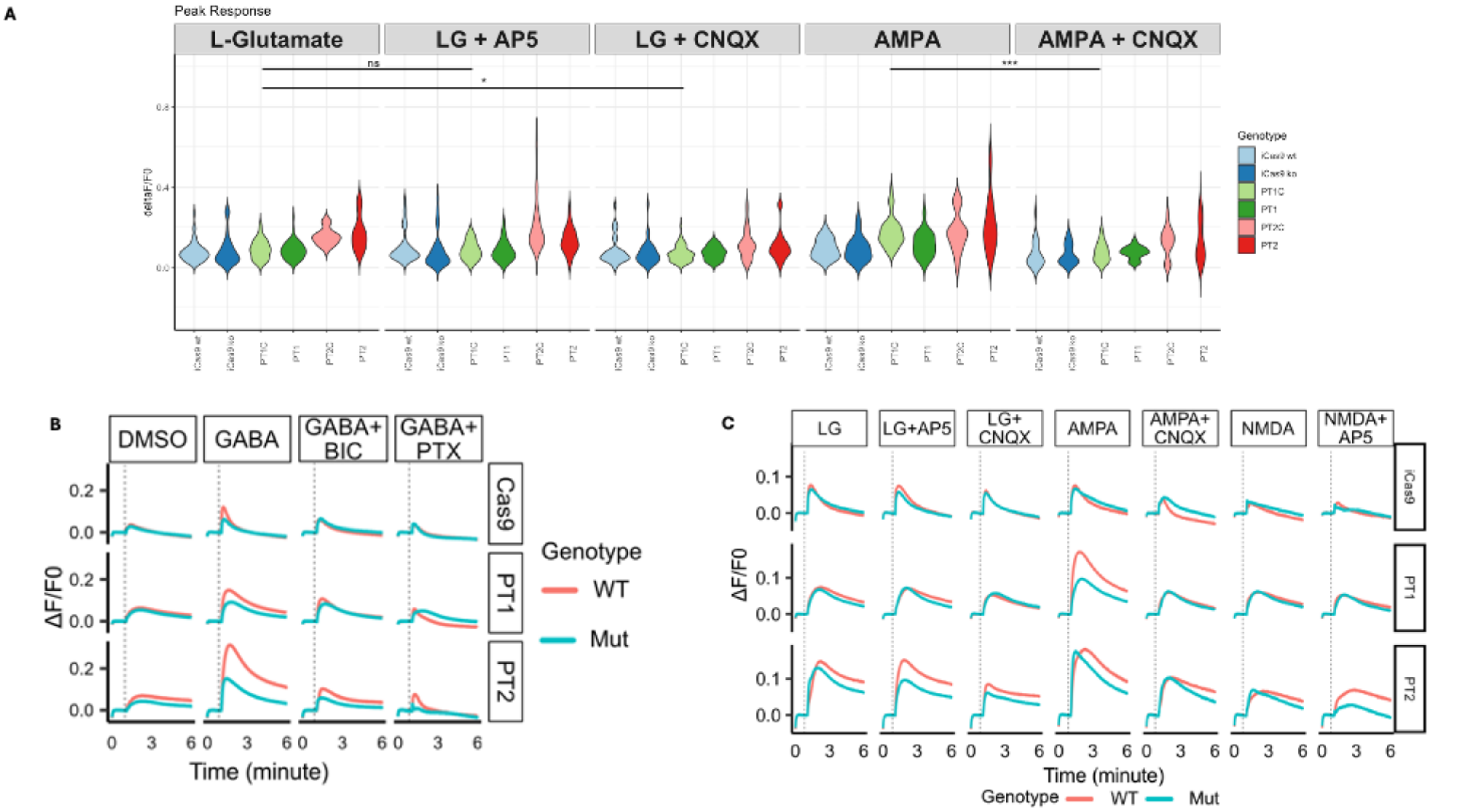

Legend: A: Violin plots demonstrating peak calcium fluorescence response ($\Delta F/F_0$) following drug injection. **B and C:** Averaged response trace ($\Delta F/F_0$) derived from the FLIPR calcium assay, with the dashed lines representing drug injection at 60 seconds for DMSO, GABA, GABA + BIC, GABA + PTX (B), and LG, LG + AP5, LG + CNQX, AMPA + CNQX, NMDA, NMDA + APF (C). Key: AMPA: α-amino-3-hydroxy-5-methyl-4-isoxazolepropionic acid; AP5: (2R)-amino-5-phosphonovaleric acid; BIC: bicuculline; CNQX: cyanquixaline; DMSO: dimethylsulfoxide; GABA: γ-aminobutyric acid; LG: L-glutamate; Mut: mutant; NMDA: N-methyl-D-aspartate; PT1: patient #1; PT2: patient #2; PTX: picrotoxin; WT: wild-type Response to small molecule application compared using one-way ANOVA. ***: $p<0.001$; **: $p<0.01$; *: $p<0.05$; ns: not significant**.**

Supplementary Figure 8: No significant difference in patch-clamp recorded passive membrane properties between *SGCE*-mutation and wild-type controls.

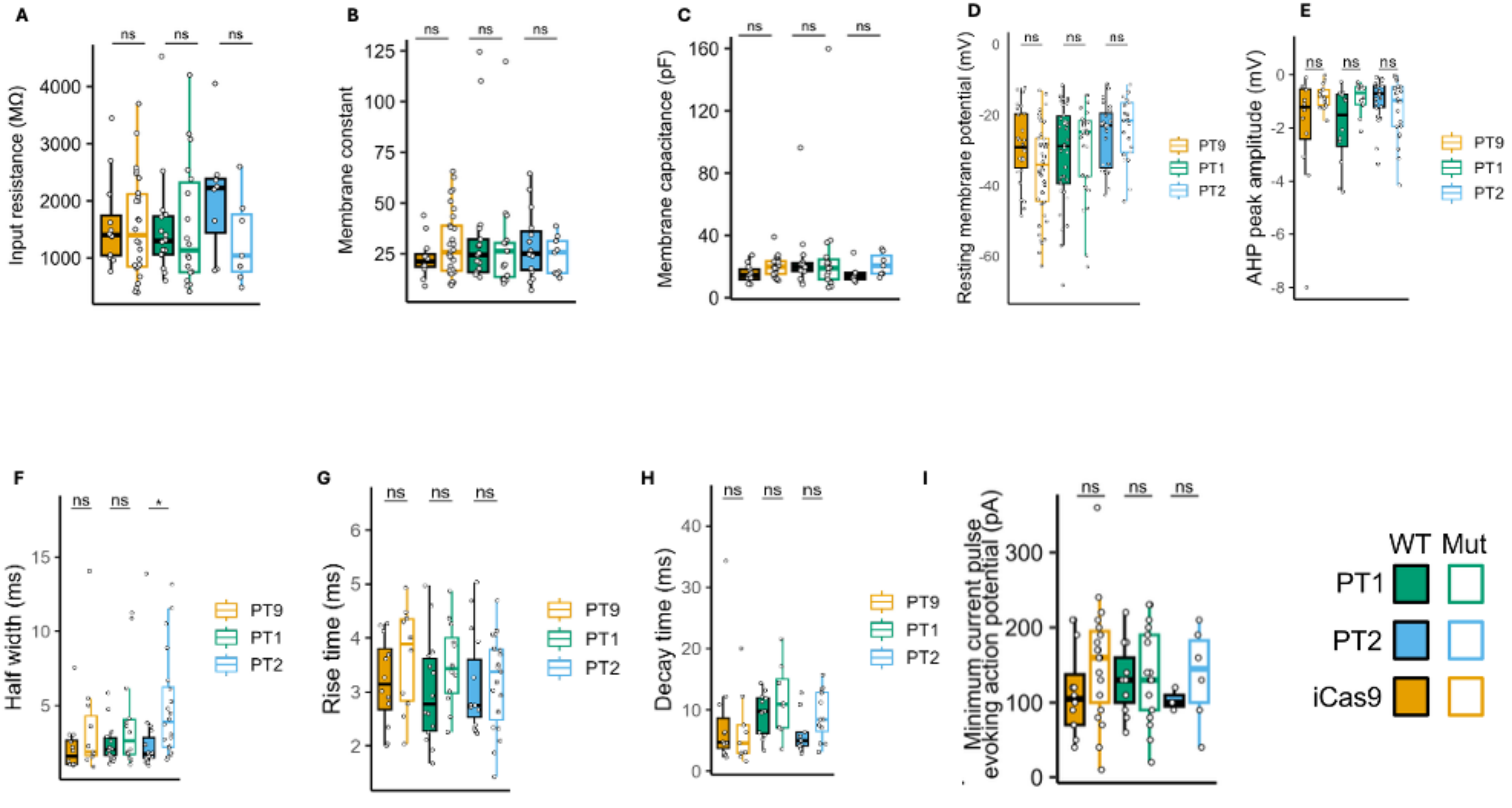

Legend: Box plots representing median and interquartile range values of passive membrane properties, comparing isogenic matched cell line pairs across measures of input resistance (**A**): Input resistance, (B): Membrane constant (**C**), Membrane capacitance (**D**), Resting membrane potential (**E**), AHP peak amplitude, (**F**), Half-width (**G**), **R**ise time (**H**), Decay time (**I**), minimum current pulse evoking action potential. Key: AHP: After-hyperpolarisation; Mut: mutant; WT: wild-type. Lines compared using Wilcoxon signed-rank tests with FDR correction ***: p<0.001; **: p<0.01; *: p<0.05; ns: not significant.

Supplementary Tables

Supplementary Table 1. Antibodies used in this study.

Name	Supplier	Catalogue Number	Dilution used
rabbit anti-CB	Swant	Cat#CB38a	1:1000
mouse anti-COUP-TFI	Perseus Proteomics	Cat#PP-H8132-00	1:1000
mouse anti-COUP-TFII	Perseus Proteomics	Cat#PP-H7147-00	1:100
rabbit anti-CR	Swant	Cat#CR7697	1:1000
goat anti-FOXA2	R&D	Cat#AF2400	1:1000
rabbit anti-FOXG1	Abcam	Cat#ab18259	1:250
rabbit anti-GABA	Sigma Aldrich	Cat#A-2052	1:1000
mouse anti-GAD67	Millipore	Cat#MAB5406	1:500
mouse anti-MAP2	Sigma Aldrich	Cat#M4403	1:500
mouse anti-NESTIN	BD	Cat#BD611659	1:1000
rat anti-NEUN-RAT	Abcam	Cat#ab279297	1:1000
rabbit anti-NKX2.1	Abcam	Cat#ab76013	1:1000
goat anti-OLIG2	R&D	Cat#AF2418	1:100
rabbit anti-PAX6-RB	Abcam	Cat#AB195045	1:1000
rabbit anti-SOX6	Abcam	Cat#ab30455	1:1000
rat anti-SST	Millipore	Cat#MAB354	1:50
donkey anti-goat IgG, Alexa Fluor 488	Invitrogen	Cat#A32814	1:1000
donkey anti-goat IgG, Alexa Fluor 555	Invitrogen	Cat#A32816	1:1000
donkey anti-goat IgG, Alexa Fluor 647	Invitrogen	Cat#A32849	1:1000
donkey anti-mouse IgG, Alexa Fluor 488	Invitrogen	Cat#A21208	1:1000
donkey anti-mouse IgG, Alexa Fluor 555	Invitrogen	Cat#A21202	1:1000
donkey anti-mouse IgG, Alexa Fluor 647	Invitrogen	Cat#A31570	1:1000
donkey anti-rabbit IgG, Alexa Fluor 488	Invitrogen	Cat#A31571	1:1000
donkey anti-rabbit IgG, Alexa Fluor 555	Invitrogen	Cat#A21206	1:1000
donkey anti-rabbit IgG, Alexa Fluor 647	Invitrogen	Cat#A31572	1:1000
donkey anti-rat IgG, Alexa Fluor 488	Invitrogen	Cat#A31573	1:1000

Supplementary Table 2. Primers used in this study.

Gene	Forward primer	Reverse primer
GAD67	CGAGGACTCTGGACAGTAGAGG	GATCTTGAGCCCCAGTTTTCTG
GAPDH	ATGACATCAAGAAGGTGGTG	CATACCAGGAAATGAGCTTG
LHX6	ACAGATCTACGCCAGCGACT	CATGGTGTCGTAGTGGATGC
NKX2.1	CGCATCCAATCTCAAGGAAT	TGTGCCCAGAGTGAAGTTTG
SST	GCTGCTGTCTGAACCCAAC	CGTTCTCGGGGTGCCATAG
